# Circadian clock components control daily growth activities by modulating cytokinin levels and cell division‐associated gene expression in *Populus* trees

**DOI:** 10.1111/pce.13185

**Published:** 2018-04-15

**Authors:** Kieron D. Edwards, Naoki Takata, Mikael Johansson, Manuela Jurca, Ondřej Novák, Eva Hényková, Silvia Liverani, Iwanka Kozarewa, Miroslav Strnad, Andrew J. Millar, Karin Ljung, Maria E. Eriksson

**Affiliations:** ^1^ School of Biological Sciences, C.H. Waddington Building University of Edinburgh Edinburgh EH9 3BF UK; ^2^ Department of Plant Physiology, Umeå Plant Science Centre Umeå University 901 87 Umeå Sweden; ^3^ RNA Biology and Molecular Physiology Bielefeld University 33615 Bielefeld Germany; ^4^ Laboratory of Growth Regulators, Centre of the Region Haná for Biotechnological and Agricultural Research Institute of Experimental Botany ASCR and Palacký University 783 71 Olomouc Czech Republic; ^5^ Department of Forest Genetics and Plant Physiology, Umeå Plant Science Centre Swedish University of Agricultural Sciences 901 83 Umeå Sweden; ^6^ Department of Statistics University of Warwick Coventry CV4 7AL UK

**Keywords:** biomass production, cell division, circadian clock, cytokinin, growth, lignification, photoperiod

## Abstract

Trees are carbon dioxide sinks and major producers of terrestrial biomass with distinct seasonal growth patterns. Circadian clocks enable the coordination of physiological and biochemical temporal activities, optimally regulating multiple traits including growth. To dissect the clock's role in growth, we analysed Populus tremula × P. tremuloides trees with impaired clock function due to down‐regulation of central clock components. late elongated hypocotyl (lhy‐10) trees, in which expression of LHY1 and LHY2 is reduced by RNAi, have a short free‐running period and show disrupted temporal regulation of gene expression and reduced growth, producing 30–40% less biomass than wild‐type trees. Genes important in growth regulation were expressed with an earlier phase in lhy‐10, and CYCLIN D3 expression was misaligned and arrhythmic. Levels of cytokinins were lower in lhy‐10 trees, which also showed a change in the time of peak expression of genes associated with cell division and growth. However, auxin levels were not altered in lhy‐10 trees, and the size of the lignification zone in the stem showed a relative increase. The reduced growth rate and anatomical features of lhy‐10 trees were mainly caused by misregulation of cell division, which may have resulted from impaired clock function.

## Abstract 2

Trees undergo annual cycles of significant primary and secondary growths. They possess an endogenous clock that enables them to adjust growth to different seasons. This study demonstrates the importance of the circa 24‐hr (circadian) clock in sustaining growth of Populus trees under diel cycles and shows the clock times expression of CYCD3 and cell division. Notably, growth hormones are disrupted in trees with broken clocks. The clock, acting via the key clock components, LHY1 and LHY2 regulates gene expression and protein function. Reducing expression of LHY1 and LHY2 reduces levels of cytokinins, which regulate plant growth, and causes a misalignment of CYCD3 expression with cell division. Together, these changes alter the balance between growth and lignification while also reducing biomass. LHY1 and LHY2 may also promote expression of BBX19 and BBX32, whose proteins appear to be part of the mechanism regulating timing of growth. Understanding clock regulation may be valuable for breeding projects targeting these important traits.

## 1 INTRODUCTION

Plants use an internal 24‐hr (circadian) clock to synchronize their metabolism and growth with predictable changes in the environment. The competitive advantage of having a clock resonating with the environmental cycle has been demonstrated in cyanobacteria (Ouyang, Andersson, Kondo, Golden, & Johnson, 1998) and the model plant *Arabidopsis* (Arabidopsis thaliana; Dodd et al., 2005; Graf, Schlereth, Stitt, & Smith, 2010; Green, Tingay, Wang, & Tobin, 2002).

The clock mechanism of *Arabidopsis* is composed of interlocked transcriptional–translational feedback loops (Millar, 2016). It resets to local time on a daily basis in response to light and temperature cues and by sensing sugar produced by photosynthesis (Haydon, Mielczarek, Robertson, Hubbard, & Webb, 2013; Shin et al., 2017). The key components of the clock include the morning‐expressed and light‐responsive MYB transcription factors CIRCADIAN CLOCK ASSOCIATED 1 (CCA1) and LATE ELONGATED HYPOCOTYL (LHY)*,* both of which repress the expression of evening genes including *TIMING OF CAB2 EXPRESSION 1* (*TOC1*/*PSEUDO‐RESPONSE REGULATOR 1* [*PRR1*]). TOC1, along with other PRR proteins (PRR7, PRR5, PRR3, and PRR9), represses expression of *CCA1* and *LHY* to complete a feedback loop (Gendron et al., 2012; Huang et al., 2012). CCA1 and LHY were originally thought to promote transcription of *PRR9* (and possibly *PRR7*) after dawn; however, recent results suggest that CCA1 and LHY instead repress these genes (Adams, Manfield, Stockley, & Carré, 2015; Fogelmark & Troein, 2014). Evening‐expressed genes including *EARLY FLOWERING 3 (ELF3*), *ELF4*, and *LUX ARRHYTHMO (LUX)*/*PHYTOCLOCK* (*PCL1*), form an evening complex (EC) that also represses components of the clock, including *PRR9* and *TOC1* (Fogelmark & Troein, 2014).

Plant growth and development are coordinated by the circadian clock. In *Arabidopsis*, this results in maximal hypocotyl elongation towards the end of the night (Nozue et al., 2007; Nusinow et al., 2011), as well as in delayed flowering under short‐day lengths (Seaton et al., 2015). *Arabidopsis*, however, is an annual species and far less is known about the regulation of growth in long‐lived plant species such as deciduous trees. The *Populus* genome contains two *LHY* genes (*LHY1* and *LHY2*), which appear to be orthologous with *Arabidopsis LHY* and *CCA1*. LHY1 and LHY2, together with TOC1, are the only proteins so far associated with clock function in *Populus* (Ibáñez et al., 2010; Takata et al., 2009). We previously showed that *LHY1* and *LHY2* are important in coordinating growth of *Populus* with the long days and warm temperatures of spring and early summer and in enabling the response to cold and the development of freezing tolerance during winter dormancy (Ibáñez et al., 2010).

Temporal regulation of growth and development may be critical in maximizing trees' fitness at high latitudes, where growing seasons are short. To understand the role of the circadian clock in maximizing biomass production in a long‐lived perennial plant, we investigated patterns of growth in trees with a faster circadian clock. We studied trees in which expression of the core clock genes *LHY1* and *LHY2* was reduced by RNAi, causing the clock period to shorten by 3–4 hr, to investigate the impact of the circadian clock in growth. To test the hypothesis that a functional clock is central for aligning daily growth processes in *Populus* trees, we carried out detailed investigations of gene expression and cell division and of metabolism of the growth regulators auxin and cytokinins, as well as of primary and secondary growth.

## 2 MATERIAL AND METHODS

### 2.1 Plant materials, growth, and sampling

All experiments were conducted using wild‐type (WT) hybrid aspen (Populus tremula × P. tremuloides) T89 cv. and *lhy‐3*, *lhy‐10*, *toc1–4*, and *toc1–5* RNAi lines, as indicated. In the RNAi lines, expression of either *TOC1* or *LHY1* and *LHY2* is reduced by ~40%, resulting in free‐running periods that are approximately 3 to 4 hr shorter than those of WT trees (Ibáñez et al., 2010). Representative RNAi lines were selected from the 10 independently derived lines described previously (Ibáñez et al., 2010).

Plants were propagated vegetatively and grown under long photoperiods (light:dark [LD] 18 hr:6 hr) at 18 °C (Ibáñez et al., 2010) or under indicated photoperiodic conditions. Nutrients (SuperbaS, Supra Hydro AB, Landskrona, Sweden) were supplied once weekly from Week 4. Plant height was measured weekly from approximately 21 days after potting. Once trees had reached approximately 20 cm in height, the stem diameters 10 cm above the soil were measured weekly.

Elongation growth rates were evaluated by a curve‐fitting procedure. Curves were fitted to the growth patterns of each plant using the linearized biexponential model (*y* = η ln [eα1(*t* − τc)/η + eα2(*t* − τc)/η] + χ; where *y*: height; η: smoothness/abruptness of the curve; α1: slope of the first linear; *t*: time; τc: constant for shifting along the *t*; α2: slope of the second linear that represents the growth rate; χ: constant for shifting along the *y*; Buchwald, 2007), using Kaleidagraph v3.6 (Synergy Software, Reading, PA, USA).

Three biological pools of leaf blade samples were collected at 4‐hr intervals from 28‐day‐old trees for microarray and metabolite analyses. Leaf material was collected from Internodes 8–11 of WT and *lhy‐10* plantlets. The 28‐day‐old trees were sampled randomly, with respect to leaf position and plant, as biological pools of leaves (one leaf per plant) collected randomly from four individual plants every 4 hr, with at least 8 hr between resampling of individual trees.

RNA for microarray analysis was obtained from two biological pools (eight plants; each pool consisted of four leaves [two leaves per tree, from two independent trees]) sampled in parallel. Sample collection started 3 hr before dawn (ZT21) and ended 48 hr later. RNA was extracted using the cetyltrimethylammonium bromide (CTAB) method (Chang, Puryear, & Cairney, 1993) and purified by an RNeasy Plant Mini Kit (Qiagen, Hiden, Germany), including DNAse treatment as described in the manufacturer's protocol and hybridized to an Affymetrix *Populus* array (Affymetrix Inc., Santa Clara, CA, USA) at the Nottingham Arabidopsis Stock Centre (NASC) array facility (Craigon et al., 2004). Gene expression profiles were confirmed in an independent experiment using quantitative reverse transcription polymerase chain reaction (RT‐qPCR). Leaves were sampled as described above; sampling began at dawn (ZT0) and ended 36 hr later. RNA was extracted and treated as described above.

Stem samples were collected from Internodes 15 and 16, as described previously (Eriksson, Israelsson, Olsson, & Moritz, 2000), at ZT1 (1 hr after lights‐on) and ZT19 (1 hr after lights‐out) using a green safelight. Samples were weighed, measured, and fixed in formaldehyde, acetic acid, and alcohol (50% ethanol, 10% formaldehyde, and 5% acetic acid) for anatomical inspection.

Auxin measurements were made on three independent pools of four leaves (biological replicates), each with three technical replicates. Material collected for cytokinin (CK) measurements consisted of a series of biological pooled samples, each with four technical replicates, collected at 4‐hr time‐points over 48 hr. The pools of leaf material collected for auxin and CK measurements overlapped with those collected for the microarray experiment.

### 2.2 Quantitative reverse transcription polymerase chain reaction

Quantitative reverse transcription polymerase chain reaction was carried out as previously described (Kozarewa et al., 2010), with an annealing temperature of 60 °C. Gene expression was normalized against expression of *ELONGATION FACTOR 1‐α* (Knight, Thomson, & McWatters, 2008). Primers for *CYCD3* (Karlberg, Bako, & Bhalerao, 2011) were based on gene model, version 3, Potri.014G023000.1.

### 2.3 Microarray analysis

Microarray data were generated by the NASC array facility using the GeneChip Poplar Genome Array (Affymetrix), with RNA from the diurnal time course sampled from WT and *lhy‐10* (as described above). Samples were processed according to NASC's standard procedure. Briefly, RNA samples were quality controlled using the Agilent 2100 Bioanalyser (Agilent Technologies, Santa Clara, CA, USA). First strand cDNA synthesis was completed using 400 units of SuperScript II Reverse Transcriptase (Invitrogen Life Technologies, Carlsbad, CA, USA) for 1 hr at 42 °C. Second strand synthesis was completed using 40 units Escherichia coli DNA polymerase I (Invitrogen), 10 units of E. coli DNA ligase (Invitrogen), and 2 units of E. coli RNase H (Invitrogen) at 16 °C for 2 hr. Following this, 10 units of T4 DNA polymerase (Invitrogen) were added to the reaction, which was incubated for a further 5 min at 16 °C before being terminated with ethylenediaminetetraacetic acid. Double‐stranded cDNA was cleaned up using the cDNA Cleanup Spin Column supplied in the Affymetrix GeneChip Sample Cleanup Module (Affymetrix) and used as a template for in vitro transcription of biotin‐labeled cRNA using the ENZO BioArray RNA Transcript Labeling Kit (Affymetrix). Biotin‐labeled cRNA was cleaned up using the cRNA Cleanup Spin Column supplied in the Affymetrix GeneChip Sample Cleanup Module and assessed for quantity and quality using the Agilent 2100 Bioanalyser (Agilent). cRNA was fragmented by metal‐induced hydrolysis to break down full‐length cRNA to 35–200 base fragments, of which 15 μg of adjusted cRNA was used to prepare 300 μl of hybridization cocktail. Two hundred microlitres of hybridization cocktail were hybridized with the GeneChip and scanned on the Affymetrix Gene Array Scanner 2500A using Micro Array Suite 5.0 software. For microarray data analysis, CEL files were preprocessed with Robust Multiarray Average (RMA) in GeneSpring version 12.5 (Agilent Technologies), in which further statistical analysis was completed. RMA preprocessing was completed using a custom generated probe mask file specific for T89 hybrid trees, which was generated according to protocols described by NASC (Graham, Broadley, Hammond, White, & May, 2007; Hammond et al., 2005), using gDNA obtained by cetyl trimethylammonium bromide extraction (Eriksson et al., 2000), and a threshold signal level of >100 was applied.

Microarray data were preprocessed with RMA in GeneSpring version 12.5 (Agilent Technologies), in which further statistical analysis was completed.

Information on *Populus* genes, including mapping of *Arabidopsis* orthologues, was obtained from version 3.0 annotations in the PopARRAY database (http://aspendb.uga.edu/index.php/databases/downloads). Array data have been uploaded to ArrayExpress as accession E‐MTAB‐4516 (https://www.ebi.ac.uk/arrayexpress/experiments/E-MTAB-4516).

### 2.4 Circadian rhythmicity scoring using COSOPT

The cosine‐wave fitting algorithm (COSOPT) analysis (without the linear regression option) was performed as described (Edwards et al., 2006) using median normalized Ln expression values exported from GeneSpring. The COSOPT method tests the fit of a single, modified cosine function with many parameters. Genes scored with a pMMC‐ß threshold of <0.05, and periods 20–28 hr were considered rhythmic (Straume, 2004). Gene Ontology analysis was carried out on clusters formed by phase‐binned COSOPT results (Edwards et al., 2006; Straume, 2004; Dataset S1). This analysis used singular enrichment analysis in AgriGO (Du, Zhou, Ling, Zhang, & Su, 2010), based on the Populus trichocarpa v3.0 annotations (PopARRAY database) and a custom background list. Genes were considered present for the analysis if at least one probe‐set represented them for each individual cluster and for the background list.

### 2.5 Bayesian Fourier clustering

Bayesian Fourier clustering analysis (Liverani, Anderson, Edwards, Millar, & Smith, 2009) was conducted using microarray data from WT trees (Dataset S2), as described previously (Edwards et al., 2006; Heard, Holmes, & Stephens, 2006). Bayesian Fourier clustering fits a wide range of waveforms, using up to five sines and cosines with a shared fundamental period.

### 2.6 CK quantification

The concentrations of endogenous CK metabolites were determined in leaves from WT and *lhy‐10* trees, sampled as described above. Extraction and purification of metabolites from 100‐mg leaf tissue or 40‐mg stem tissue samples were as described previously (Novák et al., 2003; Novák, Hauserová, Amakorová, Doležal, & Strnad, 2008). The samples were purified by combining two ion‐exchange chromatography steps (strong cation exchange, diethylaminoethyl–Sephadex combined with C18‐cartridges) with immunoaffinity purification. CK levels were quantified using ultraperformance liquid chromatography electrospray tandem mass spectrometry (Novák et al., 2008).

A mixed effects model was used to determine significant differences in levels of each metabolite between genotypes across all 13 time‐points; *p* values were calculated in R using the lme4 package (Bates, Mächler, Bolker, & Walker, 2015) with “genotype” and “time‐point” included as fixed effects and “plant” and “leaf” included as random effects.

### 2.7 Indole‐3‐acetic acid (IAA) and 2‐oxindole‐3‐acetic acid (oxIAA) quantification

The IAA and oxIAA levels were determined in leaves from WT and *lhy‐10* trees, sampled as described above. For each sample, 20‐mg plant tissue was homogenized in cold 0.05‐M sodium phosphate buffer (pH 7.0), containing 0.025% sodium diethyldithiocarbamate and labeled internal standards (^13^C_6_‐IAA and ^13^C_6_‐oxIAA). Samples were purified by solid phase extraction using mixed‐mode anion exchange sorbent (Oasis™ MAX cartridge, 1 cc/30 mg; Waters Corp., Milford, MA, USA) and injected onto a reversed‐phase column (BetaMax Neutral; 150 mm × 1 mm; particle size 5 μm; Thermo Fisher Scientific, Waltham, MA, USA) with UniGuard™ column protection (Hypurity advance; 10 mm × 1 mm; 5 μm; Thermo Scientific). Sample analyses were performed by ultraperformance liquid chromatography electrospray tandem mass spectrometry analysis using an Acquity UPLC™ System and a Quattro micro™ API mass spectrometer (Waters Corp.; Novák et al., 2012).

### 2.8 In vivo assays of promoter *CYCD3:LUCIFERASE* and *CCR2:LUCIFERASE* activities

The *CYCD3* promoter region (Potri.014G023000.1; corresponding to gene model Scaffold 961 *P_tremuloides*_ × _*P_tremula*_T89_v0001; http://popgenie.org/) was used for primer design. Nested PCR was performed to clone a 3034 bp promoter from a T89 cv. gDNA template using the following primers: First round PCR: forward 5’‐ACATCTCACCAAACTCATACAAGC‐3′ and reverse 5’‐CAGTCCTCTCTAACTTCTTCCACC‐3′; nested PCR: forward 5’‐ATAGTCGACAACGATAGGTCACATCTCTTTGGT‐3′ (Sal*I* site underlined) and reverse 5’‐ATGGATCCCTTCCAGGAAGAAGGGGTGC‐3′ (Bam*HI* site underlined; DNeasy Plant Maxi kit [Qiagen]) template.

To test the dependence of *CYCD3* expression on the *Populus* circadian clock, we cloned and fused its promoter to *LUCIFERASE* to enable real‐time analysis in WT and *lhy‐10* backgrounds. The *CYCD3* promoter sequence was ligated into *pPZP221LUC+* to produce *pCYCD3:LUC*. *pCYCD3:LUC* was introduced into WT and *lhy‐10* trees using *Agrobacterium*‐mediated transformation, as described previously (Eriksson et al., 2000), with gentamycin selection (50 μg/ml).

We also tested the dependence of the *Arabidopsis COLD*, *CIRCADIAN RHYTHM*, *AND RNA BINDING 2* (*CCR2/ATGRP*; Heintzen, Nater, Apel, & Staiger, 1997) promoter on the *Populus* circadian clock. The introduction of the *CCR2* promoter fused to *LUCIFERASE* (*CCR2:LUC*) to WT and *lhy‐10* trees has been described elsewhere (Ibáñez et al., 2010).

Levels of bioluminescence produced by the *pCCR2:LUC* and *pCYCD3:LUC* reporters were measured in detached leaves or apices of WT and *lhy‐10* plants from at least three independent lines per genotype, using one leaf from at least six different plants of each line. We entrained leaves and apices (cut and trimmed of leaves and leaf primordia) from WT and *lhy‐10* plants carrying *LUC* reporter constructs as follows: Excised tissues were placed on plates containing 0.5 × Murashige–Skoog medium (plus vitamins but without additional sucrose) and entrained to LD 18:6 photoperiods for 7 days. Tissues were then grown under LD 18:6 (equal parts blue (470 nm) and red light (660 nm) from 40 μmolm^−2^ s^−1^ light‐emitting diodes [MD Electronics]) during the light period at 22 °C. After 1–3 days, the light regime was changed at dawn (ZT0) to LL (constant red plus constant blue light) at 22 °C for recording of free‐running bioluminescence rhythms. Plant imaging data were analysed using BRASS Fourier analysis software, as described previously (Ibáñez et al., 2010). Analysis of phase was performed using data collected in LD 18:6; period length measurements were made using data collected 24–120 hr after the transfer to LL.

### 2.9 BBX, CYCD3, and LHY2 expression constructs

Coding regions of *BBX19*, *BBX32*, and *LHY2* genes were amplified from cDNA using the following primers: *BBX19* (Potri.007G015200) forward 5’‐AGAGTCGACATGCGTACCCTTTGCGACG‐3′ and reverse 5’‐GAAGGTACCGCTTTGCGATCACTCCATTAAC‐3′; *BBX32* (Potri.010G251800) forward 5’‐GAGGTCGACATGGCTGTTAAGGTTTGCGAG‐3′ and reverse GATGGTACCTCACACAGAGCACTCAGCCCA; *LHY2* (Potri.014G106800) forward 5’‐GAGGTCGACATGGAAATATTCTCTTCTGGGGA‐3′and reverse 5‐GATGGTACCGCAAGCAATATCAAGTATCAAACTG‐3′. Sal*I* sites in forward primers and Kpn*I* sites in reverse primers are underlined.

Coding regions of *BBX19*, *BBX32*, and *LHY2* were introduced into *pRT104‐3xHA* and *pRT104‐3xMyc* (Fülöp et al., 2005, Töpfer, Matzeit, Gronenborn, Schell, & Steinbiss, 1987) in frame behind the 35S Cauliflower Mosaic Virus promoter and Myc or Humaninfluenza hemagglutinin (HA)‐epitope tags. To produce CYCD3 tagged with the MYC epitope, the full coding region of *CYCD3* (Potri.014G023000.1) was obtained from *pRT104 3xMyc::CYCD3* and ligated into *pRT104‐3xHA*.

### 2.10 Protoplast protein assays

Protoplasts were prepared from an *Arabidopsis* cell culture, transfected with each pRT104 construct, and treated as described (Johansson et al., 2011). For protein stability assays, protoplasts were cotransfected with BBX19 or BBX32 and LHY2 expression constructs. After 18 hr, samples were treated with 100 μM of cycloheximide, a protein synthesis inhibitor. Samples were collected and proteins extracted at the indicated times. Protein extracts were loaded onto an 8% sodium dodecyl sulfate polyacrylamide gel and, following electrophoresis, proteins were transferred to an Immobilon‐P polyvinylidene difluoride membrane (Millipore Corporation, Billerica, MA, USA). Membranes were probed with anti‐HA antibodies to determine protein levels. To assess protein loading levels, membranes were stripped and reprobed with 1:5,000 dilution of monoclonal anti‐PSTAIR CDKA antibody (SIGMA‐Aldrich, St Louis, MO, USA).

For coimmunoprecipitation assays, transfected protoplasts were incubated for 3 hr with 50 μM proteasome inhibitor MG132 (SIGMA‐Aldrich) and then incubated with anti‐c‐Myc mouse antibody (9E10; Absolute antibody, Oxford, UK). Immunocomplexes were captured on 10 μl Protein G‐Sepharose beads, washed three times in 1 × phosphate‐buffered saline solution, 5% glycerol, and 0.2% Igepal CA‐630 buffer, and eluted by boiling in 25 μl 1 × sodium dodecyl sulfate sample buffer. The presence of BBX19, BBX32, and CYCD3 was assessed by western blotting and probing with 1:2,000 dilution of anti‐HA antibody (3F10; Roche Diagnostics, Mannheim, Germany). Finally, the beads were incubated with 1:1,000 dilution of anti‐c‐Myc chicken antibody (A21281; ThermoFisher Scientific, Waltham, MA, USA) to confirm the presence of LHY2.

Protein signals were detected following western blotting using West Femto Maximum sensitivity substrate (ThermoScientific, Rockford, IL, USA) and a FUJIFILM LAS‐3000 Luminescent Image Analyser.

### 2.11 Anatomical and biomass assays

Tissue samples were collected at ZT1 and ZT19 from Internode 16 of 119‐ and 125‐day‐old plants grown under LD 18:6, and the midinternode diameter was measured.

Samples were fixed in formaldehyde, acetic acid, and alcohol, sequentially dehydrated through a 50%, 70%, 90%, and 100% ethanol series, and embedded in LR White (TAAB Laboratories Equipment Ltd., Aldermaston, UK) in polypropylene capsules (TAAB Laboratories Equipment Ltd.). Sections 3 μm thick were cut using a Microm HM350 microtome (MICROM International GmbH, Walldorf, Germany) and heat‐fixed to glass slides. Sections were stained with toluidine blue and mounted in Entellan new (Merck KGaA, Darmstadt, Germany). Images were captured using a Zeiss Axioplan light microscope (Carl Zeiss Microscopy GmbH, Oberkochen, Germany) with an Axiocam digital camera (Zeiss). Sections were stained with phloroglucinol to visualize lignified fibres and measured. The number of cambial cells was obtained by counting 50 cambial cell files from six trees per line at each time‐point.

To visualize lignified fibres, sections from Internode 16 were stained with phloroglucinol. The extent of the lignified wood zone, identified by phloroglucinol‐staining, was measured in a blinded fashion using Metamorph (Molecular Devices, Sunnyvale, CA, USA). Wood sections were cut from six individuals of each genotype, and the width of each section was measured at 20–30 points. An average measure was calculated and normalized using the 100‐μm scale incorporated in every picture.

Plant height and diameter were measured. The volume measurement (volumetric index) was calculated as diameter^2^ × height. All remaining leaves, stems, and roots of individual plants were collected separately and weighed. The tissue was dried at 55 °C for 3 days and reweighed to determine the dry weight.

### 2.12 Statistical analyses

Statistical significance was tested using one‐way or two‐way analysis of variance (ANOVA) followed by the multiple comparisons tests or unpaired Student's *t*‐tests, as indicated, using GraphPad Prism version 6.0 for Windows (GraphPad Software, La Jolla California USA). In addition, specific statistical packages were used to analyse microarray studies, hormone measurements, and circadian rhythms, as described above.

## 3 RESULTS

### 3.1 Perturbation of the circadian clock alters growth of *Populus*

**Figure 1:**
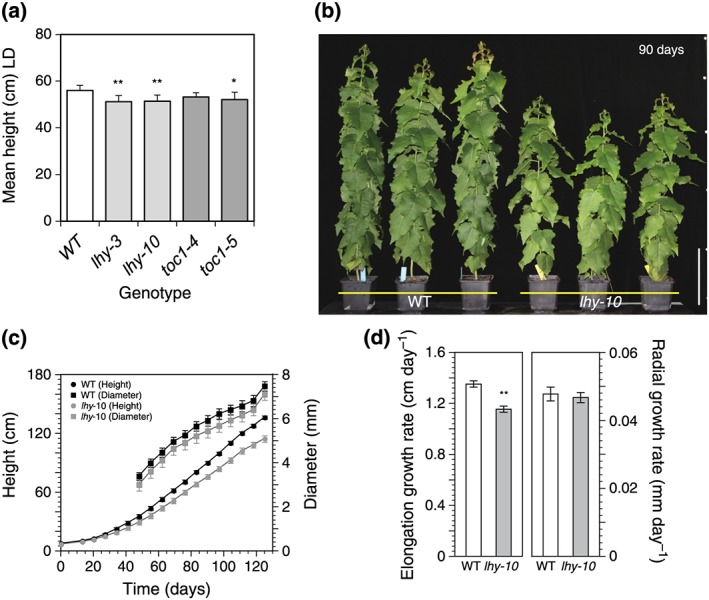
Growth of wild‐type (WT) and circadian clock RNAi trees under long days. (a) Mean height of WT and independent RNAi lines deficient in *LHY1* and *LHY2* (*lhy‐3*, *lhy‐10*) or *TOC1* (*toc1–4*, *toc1–5*) grown for 47 days under light:dark (LD) 18:6. Statistically significant growth differences detected by one‐way ANOVA followed by Dunnett's multiple comparisons test; * *p* < .05; ** *p* < .01, n = 8‐9.  (b) WT and *lhy‐10 Populus* after 90 days growth under LD 18:6. Scale bar = 20 cm. (c) Height (circles) and diameter (squares) of WT (black) and *lhy‐10* (grey) trees plotted against time over 125 days of growth under LD 18:6. (d) Rates of elongation and radial growth of 125‐day‐old WT and *lhy‐10* poplar trees. Values are means ± 1SE. **: statistically significant growth differences detected by growth curve fitting; *p* < .01; Student's *t*‐test; *n* = 15 for WT; *n* = 12 for *lhy‐10*

To investigate the impact of clock perturbations on growth in *Populus*, tree height was measured in lines which had short circadian periods due to a reduction in clock gene expression caused by RNAi. WT trees were significantly taller than the RNAi lines (Figure 1a). *lhy‐3* and *lhy‐10* had stronger growth defects (Figure 1a) and shorter internal periods (approximately 20 hr) than *toc1–4* and *toc1–5* lines (approximately 21 hr; Ibáñez et al., 2010). Heights of clock mutant trees were significantly affected: one‐way ANOVA (*p* = .0033; *n* = 8–9 per genotype) followed by Dunnett's multiple comparisons test showed that *lhy‐3* and *lhy‐10* (*p* < .01; *n* = 8–9) and *toc1–5* (*p* < .05; *n* = 8–9) but not *toc1–4* (ns; *n* = 9) differed significantly from WT. Because the clock and growth characteristics of the two *lhy* lines were similar (Figure 1a; Ibáñez et al., 2010), further investigations of height and diameter were made only in *lhy‐10* and WT trees grown under long‐day photoperiods (LD 18:6).

WT trees were larger than *lhy‐10* trees, with increased stem height and diameters (Figure 1b,c). They showed consistently greater increases in stem volumes, and higher leaf, stem, and root biomasses, with growth of *lhy‐10* being 30–40% that of WT (Tables 1 and S1; Figure S1).

**Table 1:** Measurement of stem volume, fresh, and dry weight biomass of WT and *lhy‐10* trees grown under long days

Volume (mm^3^)	WT	*lhy*‐10
Volume index	7,704.8 ± 585.6	5,995.3 ± 579.5*
Dry weight biomass (g)		
Leaf	15.5 ± 0.6	13.1 ± 0.6**
Stem	13.3 ± 1.0	9.0 ± 0.8**
Root	17.6 ± 1.2	14.2 ± 1.1*
Fresh weight biomass (g)		
Leaf	67.4 ± 4.2	58.1 ± 4.7
Stem	54.6 ± 3.8	37.8 ± 4.6**
Root	42.1 ± 2.5	32.9 ± 2.4**

*Note*. Measures are mean ± 1SE. Student's t‐test was used. WT = wild‐type; *lhy* = late elongated hypocotyl.

** Probability indicated as *p* < .01.

* Probability indicated as *p* < .05, *n* = 12 for both genotypes.

To investigate whether the perturbed growth of *lhy‐10* resulted from desynchronization between endogenous period and the environmental cycle, we measured growth under 20‐hr T‐cycles, chosen to match the internal approximately 20‐hr cycle of *lhy‐10* (Ibáñez et al., 2010). Under 10 hr light:10 hr dark T‐cycles (LD 10:10), both genotypes showed rapid growth cessation and bud set, but this response was delayed in *lhy‐10* (Figure S2a,b), consistent with their lower sensitivity to photoperiod shortening (Ibáñez et al., 2010).

To overcome the induction of dormancy, plants were grown in 16 hr light:4 hr dark (LD 16:4) T‐cycles, which supported growth of both genotypes. The daily growth rate of *lhy‐10* was approximately 80% of WT under LD 16:4 compared with 85% under LD 18:6 cycles (Figure 1 and S2c,d), thus, WT trees produced more growth even though the 20‐hr T‐cycle matched the internal period of *lhy‐10* more closely (Figure S3c,d). When growth was measured in LD 18:6 (*T* = 24 hr), WT trees grew significantly faster than *lhy‐10* (growth rates in LD: WT: 1.83 ± 0.03 cm day^−1^; *lhy‐10*: 1.69 ± 0.04 cm day^−1^ [*p* = .0093; *n* = 9]). There was, however, no significant difference in growth rates between genotypes following a shift to constant light (growth rates in LL: WT: 1.33 ± 0.04 cm day^−1^; *lhy‐10*: 1.28 ± 0.06 cm day^−1^ [*p* = .36; *n* = 9]). The growth rate of WT was reduced to the same level as *lhy‐10* in LL. All these results suggest the impaired growth of *lhy‐10* does not simply result from a mismatch between their endogenous period and the environmental LD cycle.

### 3.2 *lhy‐10* trees show reduced levels and altered metabolite profiles of CK but not IAA

**Figure 2:**
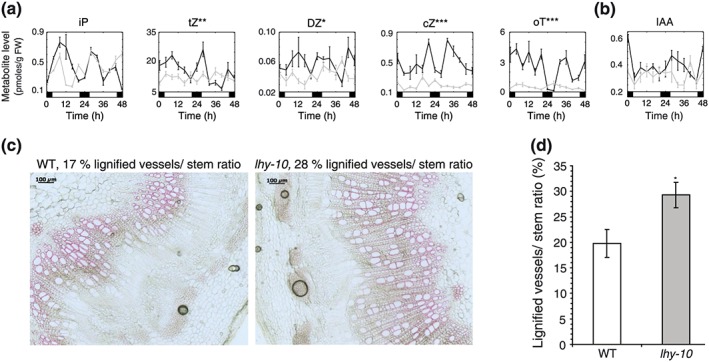
Metabolites of source leaves and lignification in young stems of wild‐type (WT) and *lhy‐10*. (a) Cytokinin metabolite levels in source leaf blades (above Internode 8–11) of WT (black) and *lhy‐10* (grey) trees grown under light:dark 18:6 for 28 days. Means are shown. (b) Indole‐3‐acetic acid (IAA) and 2‐oxindole‐3‐acetic acid metabolites in leaf blades of WT (black) and *lhy‐10* (grey) trees. Each time‐point is the mean of three biological replicates, each containing three technical replicates, ± 1SE. All measurements are pmoles/g FW. Samples were collected over 48 hr at 4‐hr intervals from ZT21 (Time 0). Asterisks indicate significant differences between genotypes according to the mixed effects model; *: *p* < .05; **: *p* < .01; ***: *p* < .001. (c) Phloroglucinol staining of 10‐μm stem sections from Internode 16 of trees grown for 125 days in light:dark 18:6. Representative images of individual WT (left‐hand side) and *lhy‐10* (right‐hand side) stem sections are shown to indicate the percentage of lignified cells. (d) Bar plot showing the lignified zone as a percentage of lignified vessels/stem ratio, based on 20–30 measurements per internode stem section of six WT and six *lhy‐10* trees. Scale bars in (a) are 100 μm, with error bars in (b) showing ±1SE. *: statistically significant difference; *p* < .05; Students *t*‐test. tZ = *trans‐*zeatin; DZ = dihydrozeatin; cZ = *cis‐*zeatin; oT = *ortho*‐topolin

Assays of auxin and CK levels in expanding source leaves after 28‐day growth in LD 18:6—before growth differences between genotypes became apparent (Figure 1c)—provided insight into the auxin status and CK metabolism of the trees. Relative to WT, CK metabolites in *lhy‐10* leaves showed substantial reductions in levels of the isoprenoid CKs *trans‐*zeatin (tZ), *cis‐*zeatin (cZ), dihydrozeatin (Sakakibara, 2006), and the aromatic *ortho*‐topolin (oT; Sakakibara, 2006; Strnad, 1997), as well as their riboside precursors tZR, cZR, oTR, and of *trans‐* and *cis‐*zeatin monophosphates (tZRMP and cZRMP, respectively; Figures 2a and S4). Levels of cZR, cZ, oTRMP, oTR, and oT dropped in WT leaves at ZT21 and rose again at dawn, possibly as a direct response to the dark to light transition.

### 3.3 Alteration in cytokinins and IAA timing and ratios separate auxin‐driven xylem differentiation and increased wood formation in *lhy‐10*

Changes in IAA levels are associated with, and required for, daily patterns of tree growth and, in particular, for cell elongation, cell division, and wood formation (Bhalerao & Fischer, 2014). Analyses of levels of IAA and its catabolite oxIAA (Pěnčík et al., 2013, Tuominen, Ostin, Sandberg, & Sundberg, 1994) in leaves showed no significant temporal or genotypic differences between lines (Figures 2b and S3), suggesting that IAA metabolism remained intact in *lhy‐10*.

We investigated the zone of lignification and xylem differentiation and found it occupied a broader area of stems in *lhy‐10* than in WT, counted as lignified vessels per area (Figures 2c and S4a,b). Phloroglucinol staining of the lignification zones in transverse sections of stem showed the extent of lignification and size distribution of fibres and vessels were similar in *lhy‐10* and WT (Figure S4c,d) but the area of lignified xylem fibres, relative to the diameter of the stem, was greater in *lhy‐10* (Figures 2d and S4b). CK metabolism and the control of auxin were thus differently affected by down‐regulation of *LHY1* and *LHY2* (Figure 2). The auxin‐related differentiation and lignification of xylem in the cambium of *lhy‐10* remained seemingly intact and, indeed, relatively expanded, and the meristem activity was more severely affected, possibly resulting from the lower levels of biologically active CK in *lhy‐10*.

### 3.4 Repression of *LHY* expression provides insights into circadian control of growth of *Populus*

**Figure 3:**
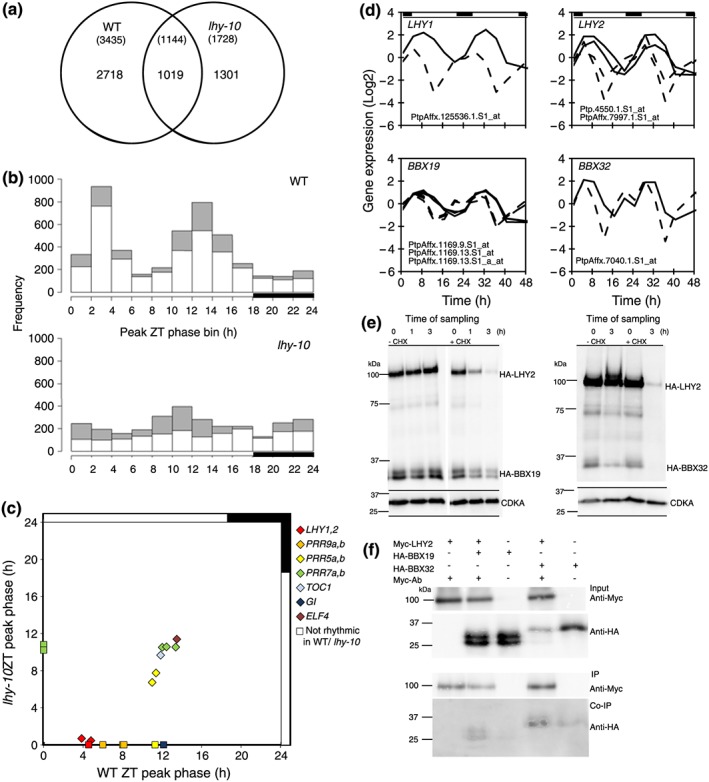
Clock‐related gene expression and protein interaction. (a–d) Microarray time‐course analyses of diurnal, rhythmic gene expression in leaf blades of 28‐day‐old WT and *lhy‐10* trees. (a) Number of rhythmic genes (number of probe sets shown in brackets) in each genotype. (b) Number of probe sets with peak expression within each 2‐hr interval across the diurnal cycle. Grey bars: total number of rhythmic genes; white bars: number of specifically rhythmic genes in a given genotype. (c) Times of peak expression of core clock gene orthologues in wild‐type (WT; *x*‐axis) versus *lhy‐10* (*y*‐axis) trees. Filled symbols: orthologues of *Arabidopsis* core‐clock genes (see inset colour key for identification); diamonds: probe sets scored as rhythmic in both genotypes; squares: probe sets scored as rhythmic in only one genotype (a phase value of 0 is assigned to the non‐rhythmic genotype). (d) Microarray time course of *LHY1*, *LHY2*, *BBX19*, and *BBX32* expression in WT (solid line) versus *lhy‐10* (dashed line) trees under light:dark 18:6. Gene acronyms and Affymetrix probe sets are shown above each plot. (e) Protein stability of HA‐tagged BBX19, BBX32, and LHY2 proteins assayed in protoplasts with and without addition of cycloheximide (CHX). Representative experiments are shown. (f) Coimmunoprecipitation following cotransfection with epitope‐tagged proteins in protoplasts. Both BBX19 (approximately 31.6 kDa) and BBX32 (approximately 34 kDa) are pulled‐down by LHY2 (approximately 93 kDa). Top panels: input levels of tagged BBX and LHY2 proteins determined using anti‐HA and anti‐c‐Myc (chicken) antibodies, respectively. Lower panels: Myc‐tagged BBX protein levels on beads were determined by immunoprecipitation (IP) with anti‐Myc antibody; bottom panel: HA‐tagged LHY2 protein levels were determined by coimmunoprecipitation (Co‐IP) with anti‐HA antibody. Representative experiments are shown; presence: +; absence: −

To investigate the effect of repressing *LHY1* and *LHY2* on circadian regulation of gene expression, we performed a microarray time‐course experiment using leaf tissue from WT and *lhy‐10 Populus* trees grown under LD 18:6. In WT *Populus*, approximately 12% of genes represented on the microarray by at least one probe set (3,737 out of 31,561 genes) showed diurnal rhythms. This fell to 7% (2,320 genes) in *lhy‐10* trees. Times of peak gene expression in WT fell into two major clusters, one centred shortly after dawn (ZT2–4) and the other before dusk (ZT12–14; Figure 3a,b). The overall distribution of phases of peak gene expression was more uniform in *lhy‐10*, and the time of peak expression in the two major temporal peaks was advanced by 2–4 hr relative to WT (Figure 3b). These changes are consistent with the short period of *lhy‐10*.

*LHY1* and *LHY2* showed peak expression around ZT4 in WT (Ibáñez et al., 2010). Their transcripts remained rhythmic in *lhy‐10* but with a 4‐hr phase advance (Figure 3c,d; Table S2). The response of the independent RNAi line *lhy‐3* resembled that of *lhy‐10*, as determined by measuring the expression of a number of representative clock‐associated genes (Figure S6; Ibáñez et al., 2010).

*Populus PRR9* orthologues had a morning phase in WT, but rhythmic expression was lost in *lhy‐10*, suggesting LHY1 and LHY2 induced *PRR9* gene expression in *Populus*. Peak expression of *PRR5* in the evening in WT trees is consistent with an evening clock‐gene role in *Populus*, and *PRR5* transcripts showed a phase advance in *lhy‐10*. Interestingly, the timing of the evening expression peaks of *Populus PRR7*s was similar in both WT and *lhy‐10*, suggesting they were less sensitive than *PRR9*s to LHY levels. As expected, both *GI* and *ELF4* showed evening phases of expression in WT (Figure 3c; Table S2; Edwards et al., 2010). Strong dusk tracking, by *ELF4* in particular, was observed, even in *lhy‐10*, which may be important for photoperiodic regulation of growth (Ibáñez et al., 2010; Nozue et al., 2007; Nusinow et al., 2011).

We used COSOPT analysis (Dataset S1) and Bayesian Fourier clustering of gene expression in WT trees (Dataset S2) to identify clusters of genes with similar expression patterns. Bayesian Fourier cluster 22 contained all three probe sets for *LHY1* and *LHY2*, together with 14 other probe sets matching 13 *Populus* gene models. This cluster contained putative homologues of circadian regulators, an ultraviolet‐ receptor and a repressor of ultraviolet‐B‐induced photomorphogenesis, as well as light and defence signalling factors. All showed moderate phase advances, suggesting clock control (Figure S5). Expression analysis of *lhy‐10* trees revealed that, although the genes in this cluster had earlier phases of expression, only two, *B‐BOX DOMAIN PROTEIN 19 (BBX19)* and *BBX32*, showed the 4‐hr phase advance that suggested a close regulatory connection with *LHY1* and *LHY2* (Figure 3d).

We hypothesized that, because LHYs and BBXs were coexpressed, they might interact in a protein complex. To test this, *Populus* BBX19, BBX32, and LHY2 proteins were overexpressed in *Arabidopsis* protoplasts, and cyclohexamide assays and coimmunoprecipitation used to investigate their turn‐over and interactions, respectively. Although all three proteins were rapidly turned over (Figure 3e), both BBX19 and BBX32 could interact with LHY2 (Figure 3f).

**Figure 4:**
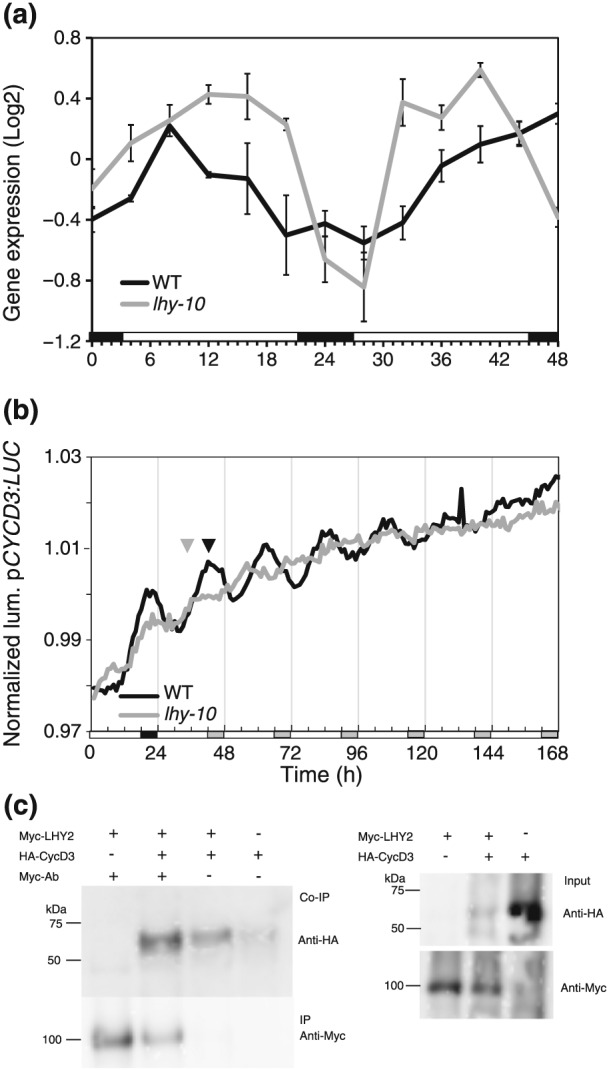
Expression of *CYCD3* is clock regulated and CYCD3 interacts with LHY2. (a) Expression of microarray *CYCD3* probe sets (Ptp.124.1.S1_at, PtpAffx.158190.1.A1_s_at, PtpAffx.60282.1.S1_s_at) in wild‐type (WT) and *lhy‐10* trees over 48 hr in light:dark 18:6 cycles. Graph shows mean expression ±1SE. (b) Normalized luminescence produced by transgenic WT and *lhy‐10* trees expressing promoter *CYCD3:LUCIFERASE* (*pCYCD3:LUC*). Luminescence was recorded in constant light (LL) after entrainment to light:dark 18:6. White and black bars indicate light and dark, respectively; white and grey bars indicate subjective day and night, respectively. Black and grey triangles indicate the phases of WT and *lhy‐10,* respectively, immediately prior to the shift to LL. (c) Coimmunoprecipitation experiments (left blot) and input protein expression (right blot) visualized using western blotting. Myc‐tagged LHY2 and HA‐tagged CYCD3 *Populus* proteins were extracted and loaded onto beads, individually or together, and with and without anti‐Myc antibody (Co‐IP. Anti‐Myc mouse antibody was used for pull‐down and anti‐Myc chicken antibodies for detection of Myc‐LHY2. A strong band (second lane from the left on left blot) shows the protein–protein interaction between Myc‐LHY2 and HA‐CYCD3 detected by anti‐HA antibodies. The input blot shows presence of protein in the samples, with the antibodies used for hybridization displayed on the right. Representative experiments are shown; presence: +; absence: −

To identify severe alterations in expression of genes associated with growth in *lhy‐10*, we applied Gene Ontology analysis to the microarray probe sets uniquely scored as rhythmic in *lhy‐10* at different times in the LD cycle. Terms associated with CK signalling and cell growth were over‐represented in the middle of the light period (ZT8–12; Dataset S1); this included genes corresponding to the growth regulator *CYCLIN D3* (*CYCD3*), which showed altered diurnal rhythmicity in *lhy‐10* (Figure 4a).

Inspection of 3034 bp of the *Populus CYCD3* promoter revealed fully conserved motifs of two CCA1‐binding elements AAMAATCT (CCA1ATLHCB1; Z. Y. Wang et al., 1997), six circadian elements CAANNNNATC (CIACADIANLELHC; Piechulla, Merforth, & Rudolph, 1998), and two evening elements AAAATATCT (EVENINGAT; Harmer et al., 2000) on either strand. In comparison, inspection of 2000 bp of the promoter of *CYCD3* genes in *Arabidopsis*; *CYCD3;1* (AT4G34160), *CYCD3;2* (AT5G67260), and *CYCD3;3* (AT3G50070; Dewitte et al., 2007), using AthaMap Webserver (Hehl & Bülow, 2014), revealed two, five, and nine predicted CCA1‐binding sites, respectively. Moreover, CCA1 was reported to bind to *CYCD3;3* by ChIP‐seq analyses (Kamioka et al., 2016).

In accordance with earlier findings (Ibáñez et al., 2010), *lhy‐10* leaves exhibited an earlier phase of *pCYCD3:LUC* expression than WT at the point of transition from LD to LL (Figure 4b). WT leaves produced a rhythmic pattern of bioluminescence in LL, whereas *lhy‐10* leaves appeared arrhythmic (Figure 4b). Period analysis revealed a mean period length of 21.39 ± 0.08 hr in WT leaves (*n* = 12 rhythmic; one arrhythmic) and that all traces (*n* = 11) from *lhy‐10* leaves were indeed arrhythmic.

**Figure 5:**
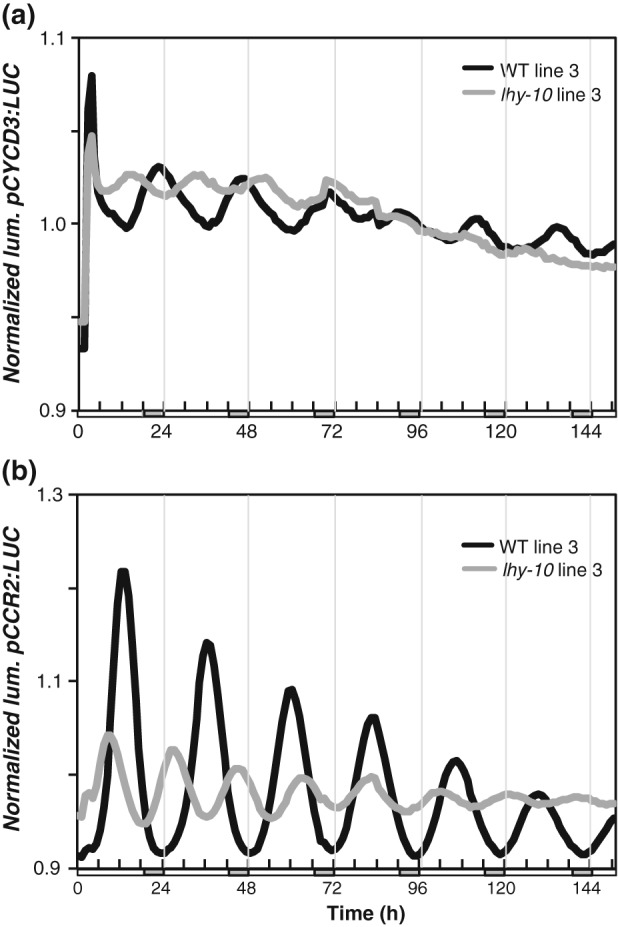
*CYCD3* and *CCR2* are clock‐regulated in an LHY‐dependent manner in stem tissues. Representative bioluminescence emitted by stem tissue from wild‐type (WT) and *lhy‐10 Populus* plants expressing promoter:reporter constructs. (a) Stem tissue expressing *pCYCD3:LUC*. (b) Stem tissue expressing *pCCR2:LUC*. Trees were entrained in light:dark 18:6 prior to imaging of cut and trimmed stem tissue under continuous light (LL). Period analyses of each line shown here and of two additional, independent transgenic lines per genotype and transgene are shown in Tables 2 and 3

We used *pCYCD3:LUC* and an additional promoter:reporter construct, *pCCR2:LUC*, to investigate the clock's performance in apices and stem tissue. Plants were initially rhythmic, although *lhy‐10* tissues had earlier phases and shorter periods than WT (Figure 5). The mean period lengths of *pCCR2:LUC* and *pCYCD3:LUC* observed in *lhy‐10* apices were 3–4 hr shorter than those of WT (Tables 2 and 3), consistent with previous observations (Ibáñez et al., 2010). Thus, *pCYCD3:LUC* is clock‐regulated, with an early phase and short period, in stem tissues of *lhy‐10* trees (Figure 5; Table 2) and has a similar pattern of expression to the well‐established evening reporter construct *pCCR2:LUC* (Figure 5; Table 3). One‐way ANOVA (*p* = .0001; *n* = 3, followed by Bonferroni's multiple comparisons test) found no significant differences between period lengths of *pCYCD3:LUC* and *pCCR2:LUC* in WT tissue (ns, *n* = 3); however, the period lengths of these reporters were significantly shorter in tissues from *lhy‐10* than in WT tissues *(p* < .0001; *n* = 3). Together, these data indicate that *CYCD3* was clock‐regulated in both apices and leaves, and dependent on LHY1 and LHY2 expression, consistent with the numerous CCA1‐binding and circadian elements present in the promoter. Thus, the disruption of circadian clock function in *lhy‐10* probably affects *CYCD3* expression directly, and this has an impact on cell division leading to diminished growth of *lhy‐10* trees.

**Table 2:** Free‐running periods measured in three independent transgenic lines carrying the reporter *pCYCD3: LUCIFERASE* (*LUC*) in wild‐type (WT) and *lhy‐10* plants under continuous light

Genotype	*pCYCD3:LUC*	Period (hr)	±1SE	Number of cuttings (rhythmic/total)
WT	Line 3 (plotted)	22.5	±0.5	8/11
	Line 5	22.6	±0.8	9/10
	Line 6	22.4	±0.4	8/11
*lhy‐10*	Line 3 (plotted)	19.4	±0.4	7/9
	Line 6	19.7	±0.5	8/11
	Line 7	19.6	±0.4	10/12

*Note*. *Populus* cuttings were grown under 18‐hr light of equal parts blue and red light/6‐h dark cycles of 40 μmolm^−2^ s^−1^ and moved to continuous light (LL) at dawn.

Analysis of free‐running (24–120 hr after transfer to LL) rhythms of *Populus pCYCD3*:*LUC* expression was performed using BRASS.

Rhythmic traces were considered to have relative amplitude error > 0.6.

Mean period calculated from three independent lines ±1SE: WT: 22.5 hr ± 0.1; *lhy‐10*: 19.6 hr ± 0.1. *lhy* = late elongated hypocotyl.

**Table 3:** Free‐running periods measured in three independent transgenic lines carrying the reporter *pCCR2: LUCIFERASE* (*LUC*) in wild‐type (WT) and *lhy‐10* plants under continuous light

Genotype	*pCCR:LUC*	Period (h)	±1SE	Number of cuttings (rhythmic/total)
WT	Line 1	22.6	±0.2	10/11
	Line 2	23.0	±0.1	9/10
	Line 3 (plotted)	23.0	±0.1	8/9
*lhy‐10*	Line 1	18.7	±0.1	10/11
	Line 2	18.6	±0.1	8/9
	Line 3 (plotted)	18.6	±0.2	7/9

*Note*. *Populus* cuttings were grown under 18‐hr light of equal parts blue and red light/6‐h dark cycles of 40 μmolm^−2^ s^−1^ and moved to continuous light (LL) at dawn.

Analysis of free‐running (24–120 hr after transfer to LL) rhythms of *Arabidopsis pCCR2:LUC* expression was performed using BRASS.

Rhythmic traces were considered to have a relative amplitude error > 0.6.

Mean period calculated from three independent lines ±1SE: WT: 22.9 h ± 0.1; *lhy‐10*: 18.7 h ± 0.1. *lhy* = late elongated hypocotyl.

### 3.5 WT and *lhy‐10* plants show different patterns of cambial activity

**Figure 6:**
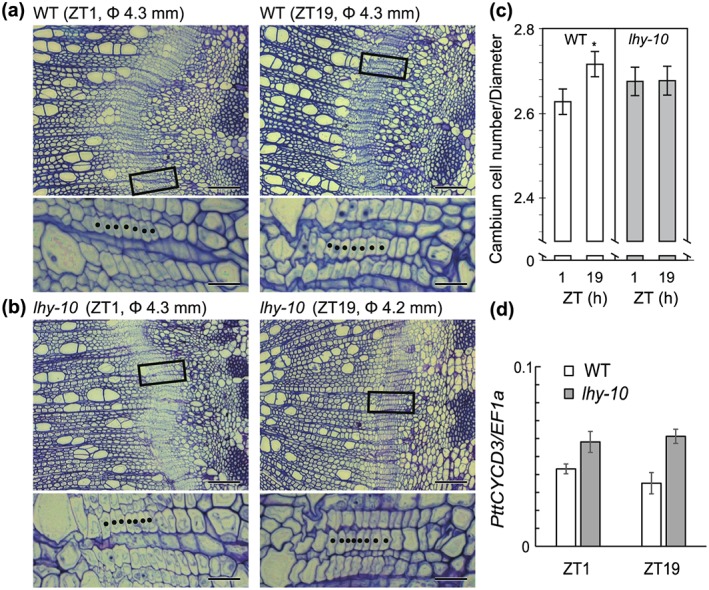
Growth changes in *lhy‐10* may be explained by deregulation of cell division activities. Representative images of toluidine blue‐stained stem cross sections from wild‐type (WT) and *lhy‐10* trees grown under long days (light:dark 18:6) taken at (a) ZT1 (b) ZT19. Identity, time, and stem diameter are indicated above each image; boxes show regions covered by the expanded views below. Closed black circles indicate cambial cells; scale bars = 100 μm (upper image) or 20 μm (lower images). Data presented in Panels (a) and (b) are from trees shown in Table S1 and Figure S4. (c) Bar plots showing ratio of cambial cell number versus stem diameter in WT (open bars) and *lhy‐10* (grey bars) trees at ZT1 and ZT19. Values are means ±1SE. *: statistically significant difference by Students *t*‐test; *p* < .05; *n* = 6. (d) Quantitative reverse transcription polymerase chain reaction determination of relative expression levels of *CYCD3* at ZT1 and ZT19 in RNA extracted from Internode 15 of WT and *lhy‐10* trees grown under similar conditions. Values are means ±1SE. Levels of gene expression were standardized against expression of *EF1α*

A major proportion of a tree's biomass is derived from activities of the cambium where cells undergo divisions and proliferation (Hertzberg et al., 2001). Our observation of premature upregulation of the cell cycle regulator *CYCD3* prompted an investigation of cambial cell activity. Observations of the cambium revealed changes in the pattern of cell division in WT and *lhy‐10* trees exposed to long photoperiods (Figures 6a–c). At ZT19, WT plants showed a higher rate of cell division than *lhy‐10*, suggesting that growth in the RNAi line was disrupted at night. Moreover, *CYCD3* expression in internodes was up‐regulated in *lhy‐10* (Figure 6d), as supported by a statistical two‐way ANOVA analysis showing a significant effect of genotype on *CYCD3* levels (*p* = .0032), but not of time (ZT; *p* = .6732) or the interaction between genotype and time (*p* = .3361) for these time‐points.

We found no significant differences in expression of *POPCORONA* (*PttPCN/PttHB5*), an orthologue of *Arabidopsis CORONA* (*CNA/ATHB15*), a gene belonging to the homeodomain‐Zip III family, which regulates secondary vascular cell differentiation and may be auxin responsive (J. Du, Miura, Robischon, Martinez, & Groover, 2011; Zhu, Song, Sun, Wang, & Li, 2013) or in expression of representative CK receptor genes *PttCRE1b* and *PttHK3a* (Nieminen et al., 2008; Figure S7). These data suggest that *CYCD3* expression and CK levels (rather than response) are directly impacted by the clock‐associated timing defect in *lhy‐10* trees, causing misalignment of its cell divisions and impairing growth.

## 4 DISCUSSION

Consideration of the circadian clock's role in regulating growth has hitherto mostly concerned the model species A. thaliana; in particular, studies of hypocotyl elongation have suggested a mechanism for the temporal control of daily growth during early development in a short‐lived annual plant. In contrast, we employed *Populus* trees to study the impact of the clock on growth in a perennial species. Perturbing *LHY1* and *LHY2* expression in *Populus* resulted in widespread changes in gene expression and a reduction in meristem activity governing stem height and diameter growth. DNA replication and mitosis are highly regulated events with major control points at G1–S and G2–M phase boundaries. “Gating” (temporally restricting) these activities so that they occur primarily at night might serve to limit DNA exposure to potentially damaging solar radiation, for instance, UVB (Takeuchi, Newton, Burkhardt, Mason, & Farré, 2014) or internal, metabolic processes generating reactive oxygen species (Wulund & Reddy, 2015). We found evidence for lower levels of radial cell division patterns in internodes of *lhy‐10* trees at night under LD cycles (Figure 6). This is consistent with altered expression of *CYCD3* in *lhy‐10* trees and interaction of CYCD3 with LHY2 (Figures 3, 4, 5, 6).

CYCD3 availability is an important rate‐limiting step for cell division in *Arabidopsis*, acting on populations of cells to maintain mitotic cycling and restrict endocycling (Dewitte et al., 2007; Menges, Samland, Planchais, & Murray, 2006). CYCD3 is receptive to mitogenic signals and functions at the G1 phase. Its expression is induced by CK in *Arabidopsis* (Riou‐Khamlichi, Huntley, Jacqmard, & Murray, 1999); however, high CK levels are not required for high *CYCD3* expression (Dewitte et al., 2007). *CYCD3* expression also regulates growth in *Populus* (Karlberg et al., 2011). Our data suggest the clock affects both CK metabolism and *CYCD3* expression, and, by generating a rhythm in *CYCD3* expression and cell division, has an important impact on growth (Figures 4, 5, and 6). As the *Arabidopsis CYCD3;1–3* promoters also contains putative CCA1‐binding sites, this mechanism of regulation by the clock may be widespread across plant species.

Of the genes scored as rhythmic in *lhy‐10*, 1,301 were specific to *lhy‐10* (Figure 3a). Although biological or technical noise cannot be ruled out, this implies that a properly functioning clock in WT trees masks or impedes rhythmic expression of a subset of genes. Interestingly, the majority of genes scored rhythmic only in *lhy‐10* showed an evening phase (ZT16–20), suggesting perturbation of *LHY* removed the clock‐based influence on dusk‐tracking transcripts in *Populus* (Figures 3b,c). Expression of these transcripts instead showed a driven rhythm matching the external LD cycle. This unmasking of strong rhythmicity in a subset of transcripts implies that, in WT trees with a fully functional clock, gene expression is buffered against temporal and environmental changes. Although peak expression of such genes showed an evening phase in *lhy‐10*, the expression of EC components did not differ greatly between *lhy‐10* and WT trees (Figure 3c), suggesting the EC does not suppress expression of this gene subset at night. Together with earlier studies (Kozarewa et al., 2010), this suggests an interaction between light and clock control, rather than conventional period resonance, underlies the timing of CK synthesis, cell division, and proliferation in *Populus*.

The 4‐hr phase advance shown by *BBX19* and *BBX32* suggested a close regulatory connection with *LHY1* and *LHY2*. Both *BBX19* and *BBX32* modulate growth in *Arabidopsis*, inhibiting photomorphogenesis (Holtan et al., 2011; C.‐Q. Wang, Sarmast, Jiang, & Dehesh, 2015). BBX19 acts as a gatekeeper of EC formation by mediating degradation of ELF3 and hypocotyl elongation (C.‐Q. Wang et al., 2015). BBX32 interacts with BBX21/STH2 to suppress ELONGATED HYPOCOTYL5 (HY5) function during light development (Holtan et al., 2011). Further, overexpression of *BBX32* sets the hypocotyl elongation phase to the dark period in *Arabidopsis* (Holtan et al., 2011) and increases yield of soybean (Glycine max; Preuss et al., 2012). Recent data from *Arabidopsis* suggest BBX32 is part of a regulatory loop with CCA1 and/or LHY, because overexpression of BBX32 increases both their expression and circadian period length (Tripathi, Carvallo, Hamilton, Preuss, & Kay, 2017). *Arabidopsis cca1;lhy* mutants, moreover, show an earlier phase of *BBX32* expression. In *Populus*, *LHY1*, *LHY2*, *BBX19*, and *BBX32* are coexpressed in WT and have a significantly earlier phase of expression in *lhy‐10* (Figure 3d), mirroring *BBX32* expression in *Arabidopsis* (Tripathi et al., 2017). Importantly, the phasing of *BBX19* and *BBX32* may be part of a clock‐associated growth control mechanism across diverse species, including *Arabidopsis,* soybean and *Populus* (Figure 3).

Stem elongation, cell division, and wood formation in *Populus* are shaped by the interactions of multiple plant hormones, including auxins, CKs, ethylene, and gibberellins (Eriksson et al., 2000; Israelsson et al., 2003; Love et al., 2009; Nieminen et al., 2008; Tuominen, Puech, Fink, & Sundberg, 1997). Auxin and CKs are exported from actively growing “source” leaves and act as mobile signals regulating cell division in the meristems of the apices, cambium, and roots (Nieminen et al., 2008). Whereas the bioactivities of dihydrozeatin, immunoprecipitation (IP), and tZ are well‐known (Sakakibara, 2006), those of cZ and oT are less well established. However, the *Arabidopsis* HISTIDINE KINASE 4 (AHK4/CRE/WOL) receptor responds to tZ and the aromatic CK, *meta‐*topolin. Similarly, the Zea mays HISTIDINE KINASE 1 (ZmHK1) receptor responds to cZ and oT, which suggests all these species are active CKs (Mok et al., 2005). The reduction in levels of CK metabolites (and especially of tZ, cZ, and oT) in *lhy‐10* trees suggested their reduced growth rate might result from a change in CK metabolism. The growth reduction observed in *lhy‐10* phenocopies that of transgenic trees in which levels of active CKs are reduced by an increase in CYTOKININOXIDASE2 (CKX2) expression (Nieminen et al., 2008). Both CK levels and the diurnal pattern of metabolites are likely to be responsible for driving growth. Our data indicate CK metabolism in leaves is under both circadian and diurnal controls (Figures 2a and S4).

**Figure 7:**
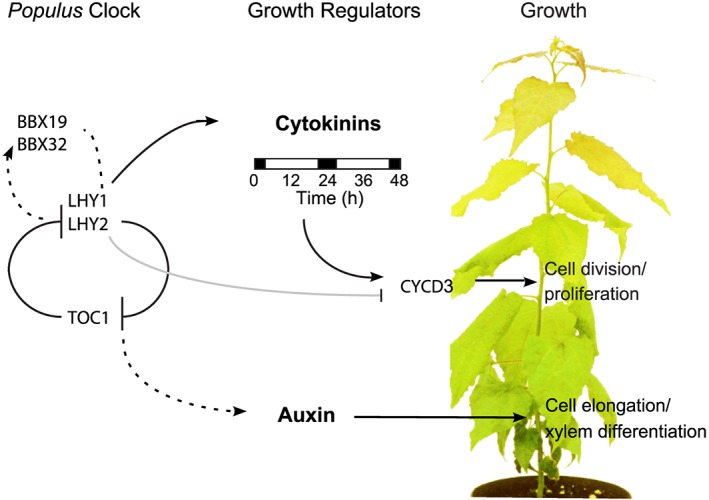
Temporal dissection of regulators of plant growth and development. Overview of growth coordination by the *Populus* oscillator. Cytokinin biosynthesis is controlled and sustained during the day. High levels of bioactive cytokinins are known to control cell division and proliferation. Environmental cues such as light and temperature reset the clock to local time. During the day, the clock, acting *via* LHYs, regulates *CYCLIN D3* gene expression and protein function. LHY1 and LHY2 may promote expression of *BBX19* and *BBX32* and their proteins may be part of the timing mechanism regulating growth. Other components (Ibáñez et al., 2010; Takata et al., 2009), dependent on, for instance, TOC1, may maintain or respond to auxin and act to promote elongation growth similar to *Arabidopsis* hypocotyls (Covington & Harmer, 2007), xylem differentiation, and lignification (Bhalerao & Fischer, 2014). Tentative interactions are indicated as dashed lines

Auxin plays an important role in cell division, and a gradient of auxin over the cambium regulates secondary cell wall biosynthesis (Hertzberg et al., 2001; Tuominen et al., 1997; Uggla, Moritz, Sandberg, & Sundberg, 1996). Importantly, auxins and cytokinins function in different parts of the stem to regulate cell division and xylem differentiation; cytokinins have been specifically shown to affect diameter growth (Immanen et al., 2016). Although auxin biosynthesis, xylem formation, and lignin biosynthesis appeared normal in *lhy‐10*, the relative amount of wood laid down was altered (Figure 2). The opposite effects seen on plant growth and secondary cell wall formation indicate CK biosynthesis is temporally and/or spatially separated from the clock's control of auxin in *lhy‐10* (Figures 1 and 2). Together with the observed patterns in hormone cycles, this suggests the clock differentially regulates the auxin and CK systems. Although the phases of *LHY* and *PRR9* expression were strongly affected in *lhy‐10*, expression of most EC members (including *ELF3*, *ELF4*, and *LUX*) was similar in WT and *lhy‐10* (Table S2), and *ELF4* remained rhythmic in *lhy‐10* with only a small phase advance (Figure 3; Table S2). This is consistent with regulation of diurnal auxin levels by the EC in *Populus* (Figure 7).

Biomass, defined as wood volume, is negatively correlated with lignin content (Novaes, Kirst, Chiang, Winter‐Sederoff, & Sederoff, 2010). Rational manipulation of this trade‐off requires biological regulators that dissect growth and wood development. The clock provides one such target regulator because of its distinctive, rhythmic control of plant metabolism and growth; for instance, *PRR7a* and *ELF3* are candidate genes for *Populus* QTLs underlying diameter growth and internode count, explaining 4.42% and 6.69% of genetic variation, respectively (Novaes et al., 2009).

We propose that maximal expression of LHY1 and LHY2 during the morning promotes growth by activating genes such as *BBX19* and *BBX32* and that this affects CK biosynthesis and responses. These features may be linked; up‐regulation of BBX32 in soybean gives a “stay green” phenotype consistent with high CK levels (Preuss et al., 2012), and CK perception and response proteins interact with *Arabidopsis* BBX32 (Tripathi et al., 2017). Our findings match reports of daily leaf growth rhythms in *Populus deltoides,* which shows high rates of growth during the evening and night (Matsubara et al., 2006). Reports from green algae (*Chlorella* sp.), flagellate algae (Euglena gracilis), and cyanobacteria (*Synechococcus* sp.) suggest the circadian clock gates cell division and growth (Bolige, Hagiwara, Zhang, & Goto, 2005; Mori, Binder, & Johnson, 1996; Stirk et al., 2011), with the level of active CKs affecting this gating (Stirk et al., 2011). Moreover, cyclins and *Wee1* have been implicated in the circadian control of mammalian cell division (Feillet et al., 2014; Matsuo et al., 2003).

Our study is a first step towards understanding the clock's multilayered, temporal control of growth and biomass production in a perennial tree species. We show that the circadian clock acts to regulate cell division, probably through control of *CYCD3* expression and physical interactions between proteins. This suggests a close interregulatory link**,** between the circadian clock and the cell cycle which is fundamental to the control of growth and production of biomass.

## AUTHOR CONTRIBUTIONS

Conceptualization: K. D. E., N. T., A. J. M., and M. E. E. Methodology: K. D. E., N. T., K. L., M. S., A. J. M., and M. E. E. Investigation: I. K., N. T., M. Jo., M. Ju., O. N., E. H., M. S., K. L., and M. E. E. Formal analysis: K. D. E., M. Jo., N. T., S. L., M. E. E. Resources: Writing—original draft: K. D. E., A. J. M. and M. E. E. Writing—review & editing: M. E. E. Supervision: M. E. E. Project administration: A. J. M. and M. E. E. Funding acquisition: A. J. M. and M. E. E.

## CONFLICT OF INTEREST

M. E. E. is a member and board member (CEO) of the holding company Woodheads AB, a part‐owner of SweTree Technologies, which played no part in this work. In addition, a patent application (WO2014087159) related to findings reported in this manuscript has been submitted.

## Supporting information

Data S1 Supporting information
