# Supplementary material for: Circadian clock components control daily growth activities by modulating cytokinin levels and cell division‐associated gene expression in Populus trees
Source: Plant Cell Environ. 2018 Apr 15;41(6):1468–82. doi: 10.1111/pce.13185 (PMC6001645; doi:10.1111/pce.13185)
Supplement: Supplementary file 1 — Data S1 Supporting information [file PCE-41-1468-s001.zip › FigS1_05_April.pdf]

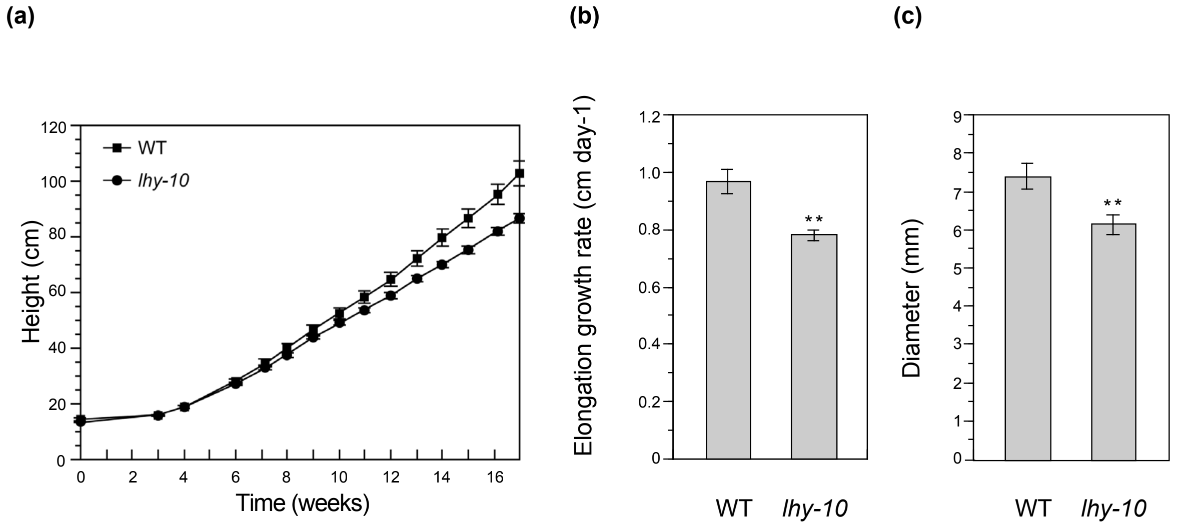

Figure S1.
(a) Plot showing tree height over 17 weeks (119 days) growth in WT and *lhy-10* trees under long days (18 h light/ 6 h dark). See inset key for identification. (b) Average elongation growth rate and (c) stem diameter shown for trees after 119 days of growth of WT and l*hy-10* mutant *Populus* trees. All values are means ± standard error (± 1SE). Statistically significant difference based on a Student's t-test **, $P < 0.01$, n=12.
