# Supplementary material for: Circadian clock components control daily growth activities by modulating cytokinin levels and cell division‐associated gene expression in Populus trees
Source: Plant Cell Environ. 2018 Apr 15;41(6):1468–82. doi: 10.1111/pce.13185 (PMC6001645; doi:10.1111/pce.13185)
Supplement: Supplementary file 1 — Data S1 Supporting information [file PCE-41-1468-s001.zip › FigS2_05_April.pdf]

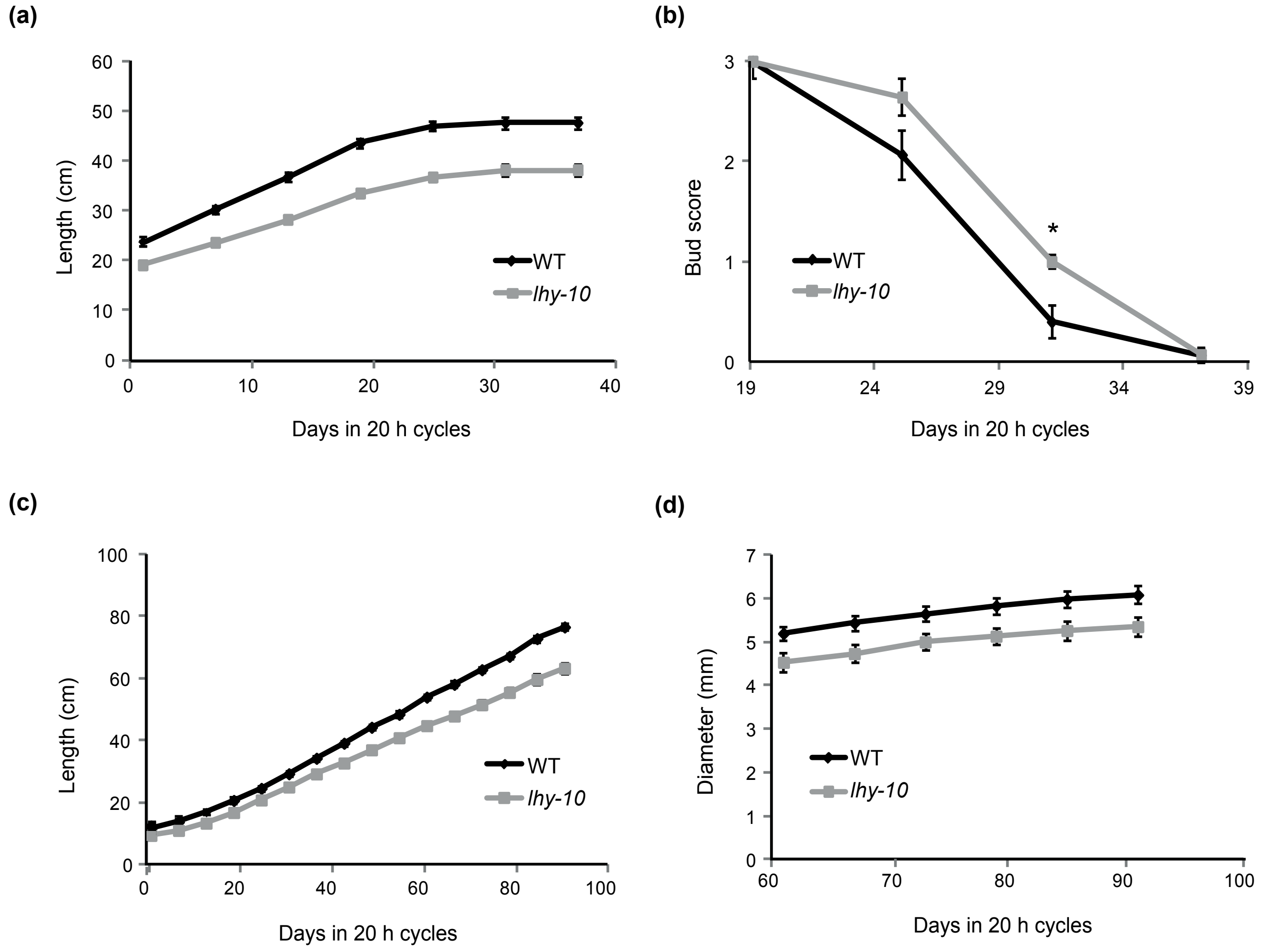

Figure S2.
WT grow better than *lhy-10* trees under T-cycles.
Height growth (a) of WT and *lhy-10 Populus* plants after growth in 20h days (10:10; 10-hour light and 10-hour dark cycles, 18°C constant temperature and 80% humidity). Error bars denote 1SE. P= 0.01 or lower for all measured time points. n = 15 (WT), n = 14 (*lhy-10*).
(b) shows scoring of bud set for the same population. Error bars denote 1SE. Significant differences are noted by *, $P < 0.05$.
Height growth (c) and diameter growth (d) of WT and *lhy-10 Populus* plants grown in 20 h days, but with 16-hour light and 4-hour dark cycles (16:4). Error bars denote 1SE. P= 0.01 or lower for all measured time points, n = 14 (WT), n = 10 (*lhy-10*).
