# Supplementary material for: Circadian clock components control daily growth activities by modulating cytokinin levels and cell division‐associated gene expression in Populus trees
Source: Plant Cell Environ. 2018 Apr 15;41(6):1468–82. doi: 10.1111/pce.13185 (PMC6001645; doi:10.1111/pce.13185)
Supplement: Supplementary file 1 — Data S1 Supporting information [file PCE-41-1468-s001.zip › FigS3_05_April.pdf]

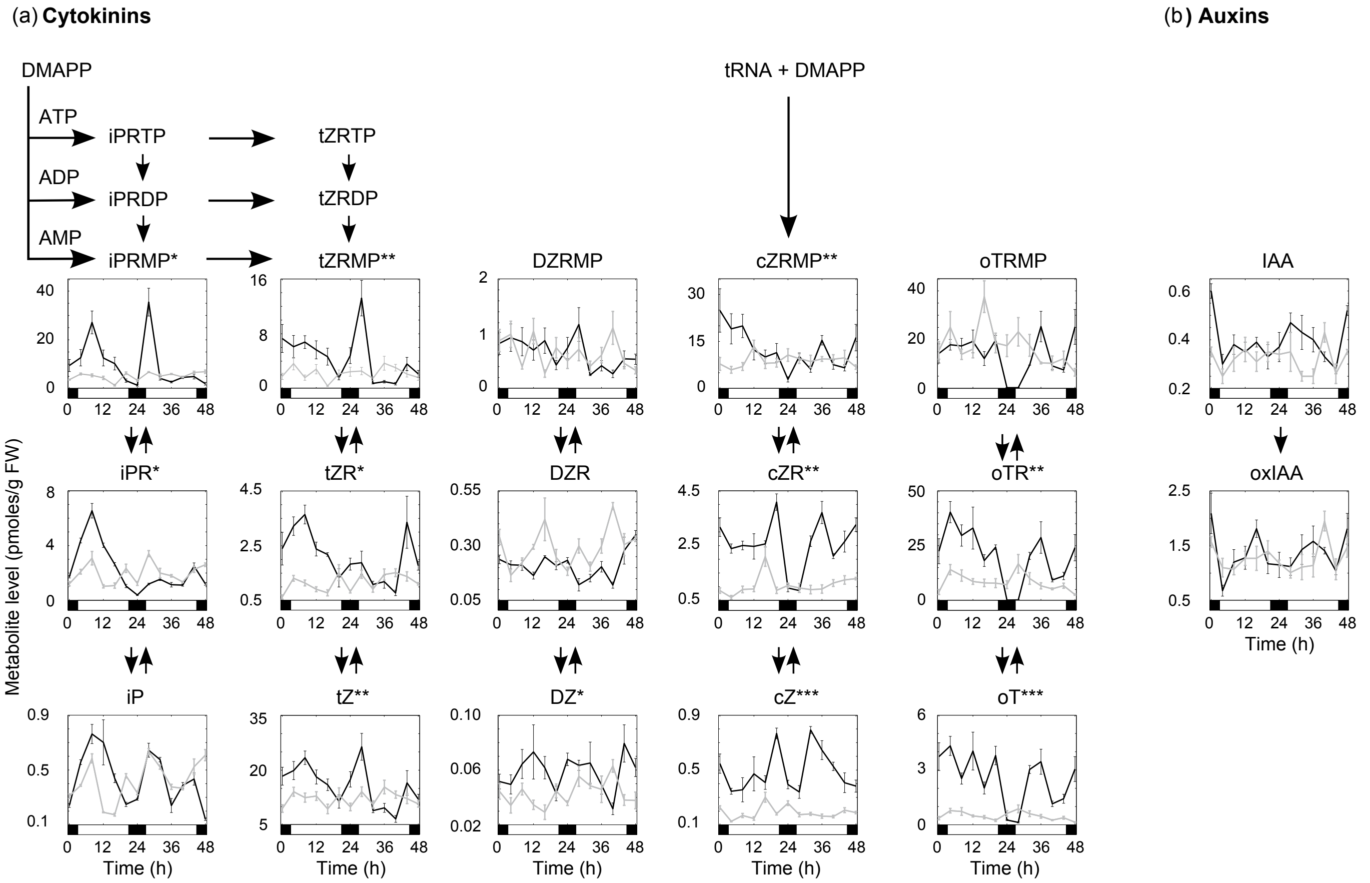

Figure S3.
Full metabolite profiles detected in WT and *lhy-10* trees (parts of which shown in Figure 2)
(a) Levels of cytokinin metabolites in leaf blades of wild type (black) and *lhy-10* (grey) trees. Samples from four randomly selected *Populus* trees per genotype were pooled at each time point. Arrows indicate the direction of biosynthesis and degradation. Mean values are shown. Error bars in (a) represent ± 1SE from one biological pool with four technical replicates.
(b) IAA and IAA-ox metabolites measured in leaf blades of wild type (black) and *lhy-10* (grey) trees.
Each time-point shows the mean value of three biological replicates, each containing three technical replicates.
Sampling starting at ZT 21 (time 0).
All measurements show pmoles/g FW. Significant differences of metabolites following statistical Mixed effects models showing significant effects for Genotype (*P < 0.05, ** P < 0.01, *** P < 0.001) on the level of each metabolite represented within each plot.
p values were calculated in R using lme4 package including plant and leaf as random effects as described in ‘Material and Methods’.
