# Supplementary material for: Circadian clock components control daily growth activities by modulating cytokinin levels and cell division‐associated gene expression in Populus trees
Source: Plant Cell Environ. 2018 Apr 15;41(6):1468–82. doi: 10.1111/pce.13185 (PMC6001645; doi:10.1111/pce.13185)
Supplement: Supplementary file 1 — Data S1 Supporting information [file PCE-41-1468-s001.zip › FigS4_05_April.pdf]

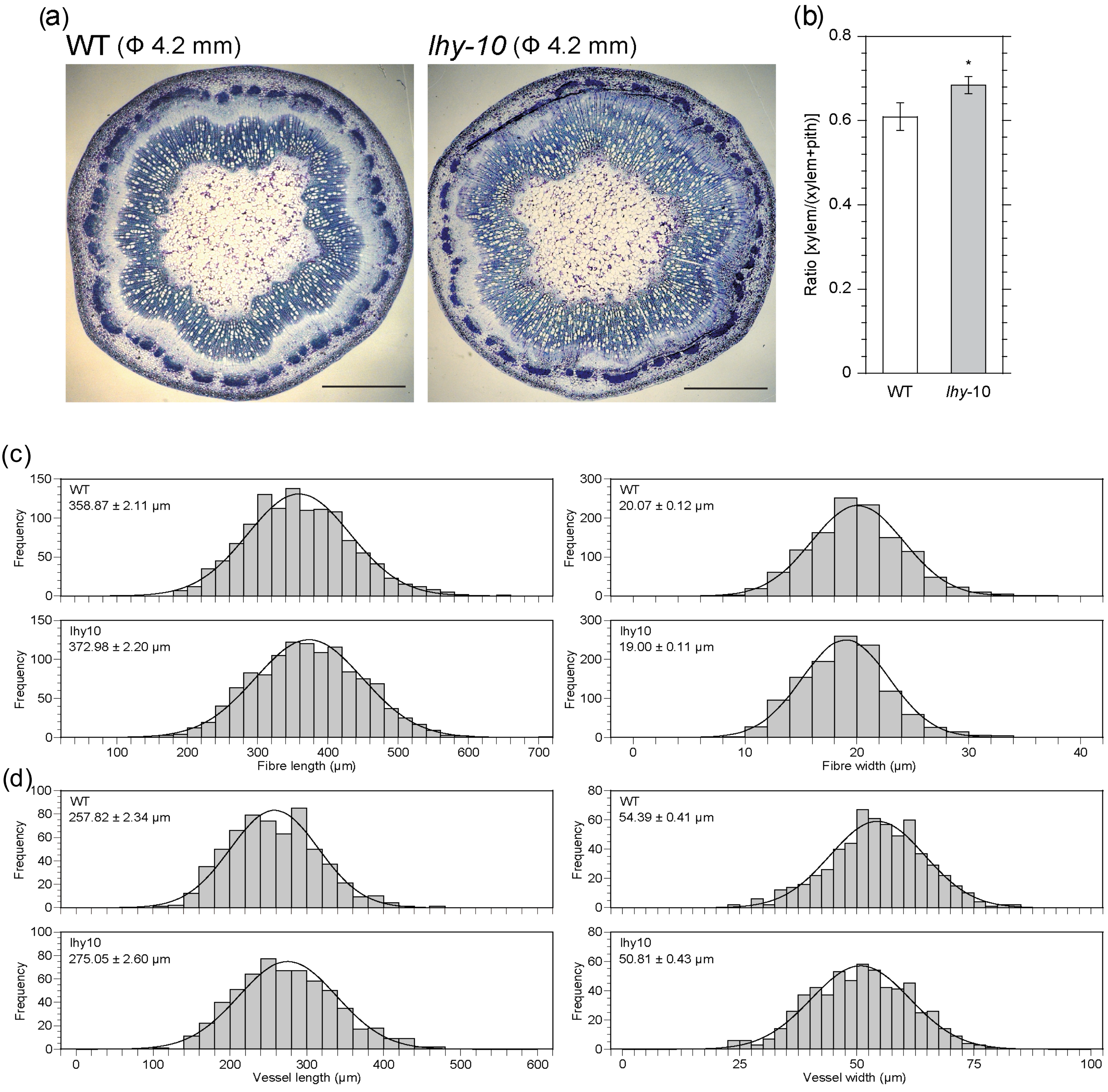

Figure S4.
Overview of stem sections stained with toluidine blue-O reveal an altered ratio of xylem differentiation (upper panel). Frequency distribution of xylem fibre and vessel lengths and widths appear normal (lower panels).
(a) Toluidine blue-O stem sections of WT and *lhy-10*. Genotypes and stem diameter are indicated above each image. Scale bar = 1 mm. (b) Relative ratio of xylem area to xylem and pith area combined. Areas were measured using ImageJ software. Values are means ± 1SE. Statistically significant differences by Student's t-test *, $P < 0.05$.
Frequency distribution of (c) xylem fibre and (d) xylem vessel lengths and widths in WT and *lhy-10.* Stem samples from internode 15 were macerated in a solution of 30 % hydrogen peroxide and glacial acetic acid (1:1) at 95°C for six hours. Xylem cells were observed by a Zeiss Axioplan light microscope, length and width of xylem fibre and vessel cells were measured using Axiovision 4.5 software (Zeiss). Two hundred fibre cells and hundred vessel cells were estimated from six trees per each genotype. Values are means ± 1SE.
