# Supplementary material for: Circadian clock components control daily growth activities by modulating cytokinin levels and cell division‐associated gene expression in Populus trees
Source: Plant Cell Environ. 2018 Apr 15;41(6):1468–82. doi: 10.1111/pce.13185 (PMC6001645; doi:10.1111/pce.13185)
Supplement: Supplementary file 1 — Data S1 Supporting information [file PCE-41-1468-s001.zip › FigS5_05_April.pdf]

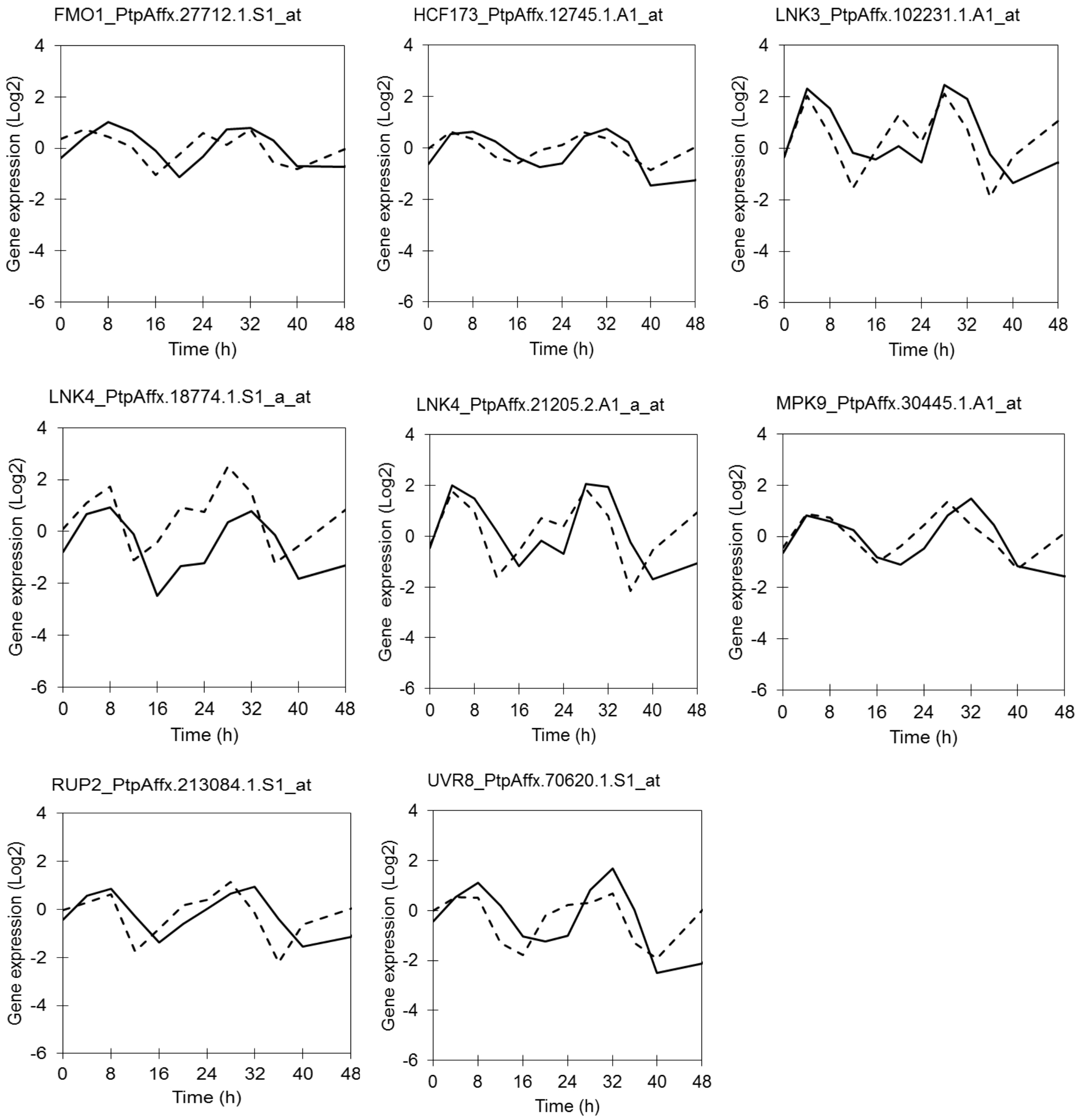

Figure S5.
Co-expressed genes in cluster 22 mainly associated with the circadian and UV-light reception and response.
Micro array expression of gene models putatively corresponding to homologues in Populus are shown for WT (solid line) and lhy-10 (dashed line) trees. Mean expression of probe sets are plotted over the course of the microarray experiment. Gene acronyms and Affymetrix probe sets are shown above each plot,Y-axes show gene expression levels as median normalised Log2 values and X-axes show experiment time starting sampling at ZT 21 (time 0) under long days of 18 h light: 6 h dark.
Micro array expression values are shown for FLAVIN-DEPENDENT MONOOXYGENASE 1 (FMO1), HIGH CHLOROPHYLL FLUORESCENCE PHENOTYPE 173 (H173), NIGHT LIGHT-INDUCIBLE AND CLOCK-REGULATED 3 (LNK3), LNK4, MAP KINASE 9 (MPK9), REPRESSOR OF UV-B PHOTOMORPHOGENESIS 2 (RUP2), UVB-RESISTANCE 8 (UVR8).
