# Supplementary material for: Circadian clock components control daily growth activities by modulating cytokinin levels and cell division‐associated gene expression in Populus trees
Source: Plant Cell Environ. 2018 Apr 15;41(6):1468–82. doi: 10.1111/pce.13185 (PMC6001645; doi:10.1111/pce.13185)
Supplement: Supplementary file 1 — Data S1 Supporting information [file PCE-41-1468-s001.zip › FigS6_26_Dec_3.pdf]

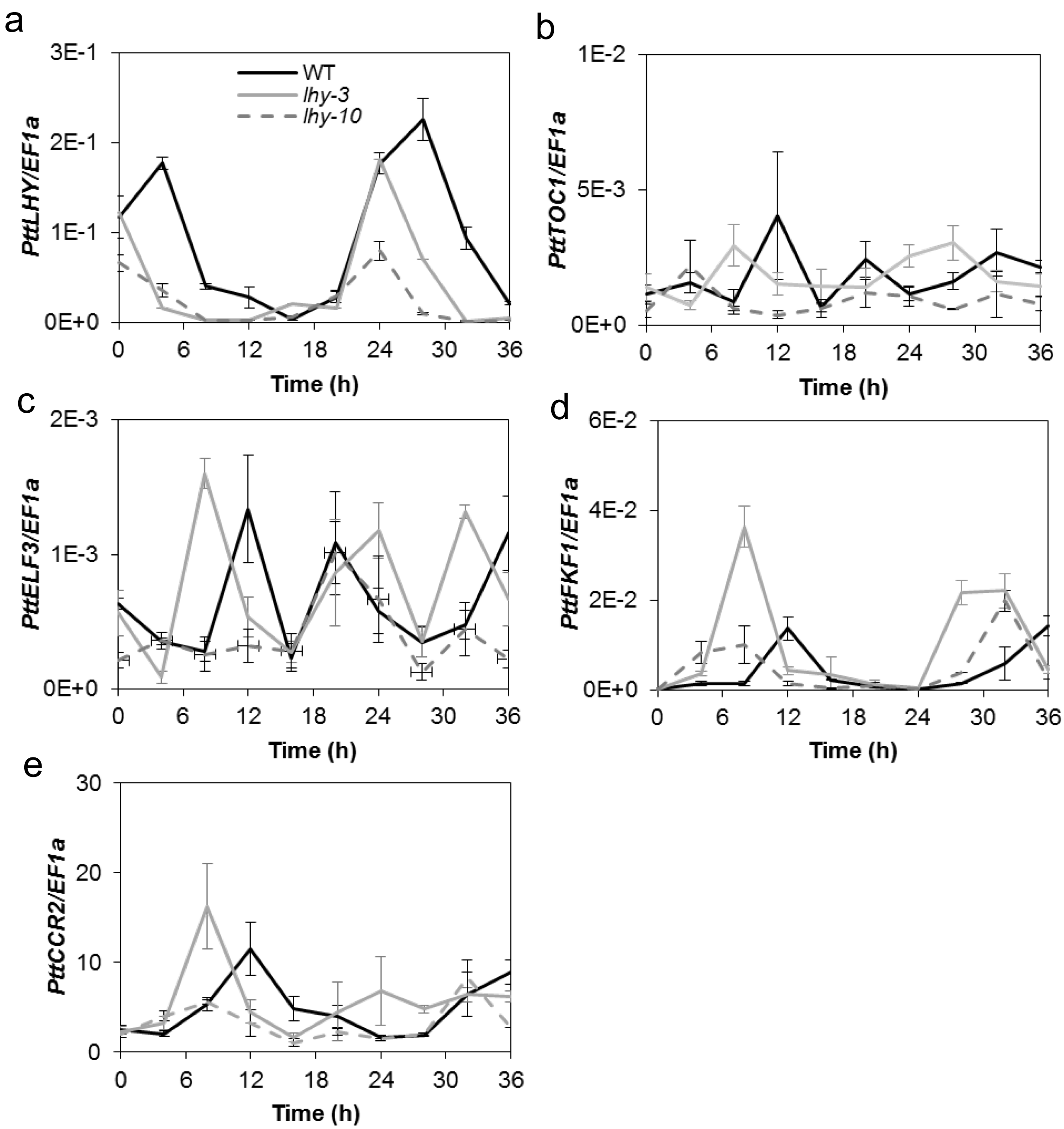

Figure S6.
Relative gene expression of clock-associated and a photoperiodic out-put genes under long days, 18:6 L:D.
Leaves from independent plants were randomly sampled and pooled from WT and *lhy-10* trees respectively, subsequently processed and used as template for RT-qPCR. Expression relative the reference gene EF1α is shown for clock-associated genes (a) *PttLHY1* and *PttLHY2* combined, (b) *PttTOC1*, (c) *PttELF3*, a clock controlled photoperiodic gene, (d) *PttFKF1* and the clock regulated, slave oscillator gene (e) *PttCCR2*. All values are means of three technical replications ± 1SE, time points are shown as time (h) of sampling relative dawn day 1, which started at dawn (ZT0). Lights-off occured at 18 h after dawn.
