# Supplementary material for: Circadian clock components control daily growth activities by modulating cytokinin levels and cell division‐associated gene expression in Populus trees
Source: Plant Cell Environ. 2018 Apr 15;41(6):1468–82. doi: 10.1111/pce.13185 (PMC6001645; doi:10.1111/pce.13185)
Supplement: Supplementary file 1 — Data S1 Supporting information [file PCE-41-1468-s001.zip › FigS7_26_Dec.pdf]

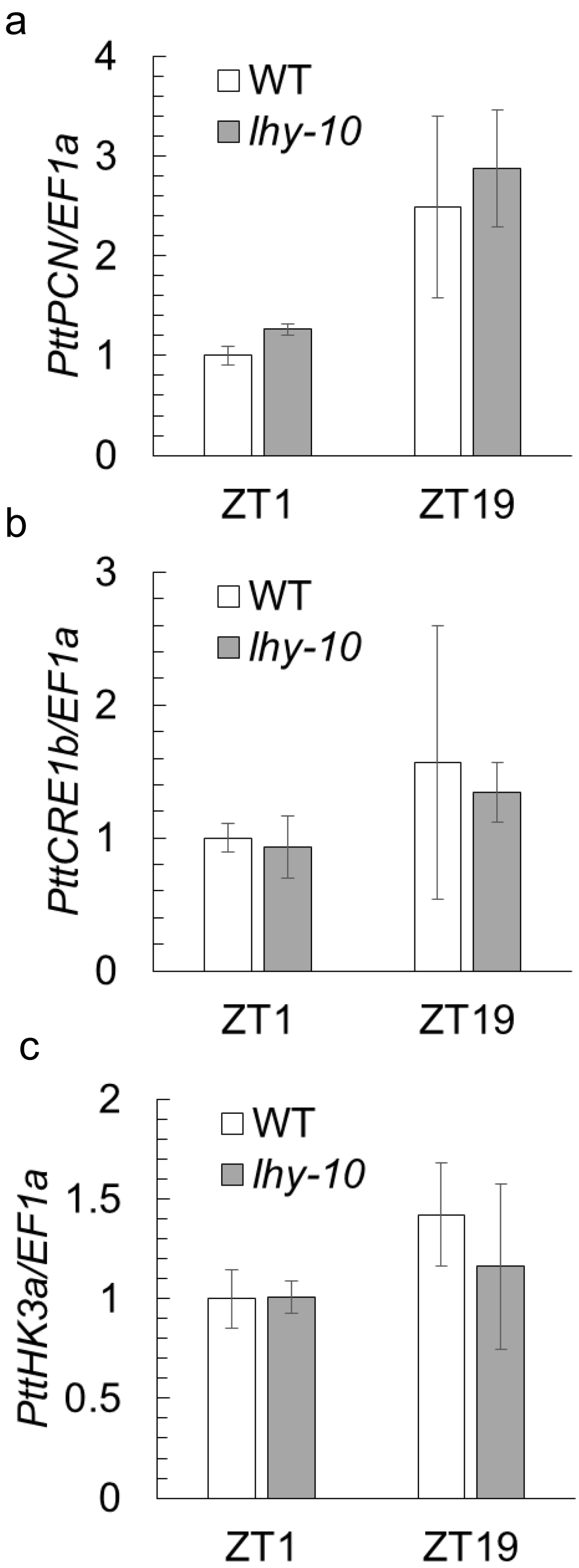


Figure S7.
RT-qPCR determination of relative expression levels of (a) *PttCNA,* (b) *PttCRE1* and (c) *PttHK3a* at ZT1 and ZT19 in RNA extracted from internode 15 of WT and *hy-10* trees grown under long days 18:6 L:D. Values are means ± 1SE (n = 3–4). Levels of gene expression were standardized unsing the reference gene EF1α.
