# Supplementary material for: Circadian clock components control daily growth activities by modulating cytokinin levels and cell division‐associated gene expression in Populus trees
Source: Plant Cell Environ. 2018 Apr 15;41(6):1468–82. doi: 10.1111/pce.13185 (PMC6001645; doi:10.1111/pce.13185)
Supplement: Supplementary file 1 — Data S1 Supporting information [file PCE-41-1468-s001.zip › Table S1.pdf]

Table S1. WT have larger volume and accumulate more biomass than *lhy-10* trees

**Volume (mm^3)**	WT	*lhy*-10
Volume index	5783.7 ± 684.8	3422.6 306.5**
**Dry weight biomass (g)**		
Leaf	8.9 ± 1.0	5.0 ± 0.4**
Stem	7.3 ± 0.9	4.0 ± 0.4**
Root	5.5 ± 0.7	3.2 ± 0.4**
**Fresh weight biomass (g)**		
Leaf	40.3 ± 4.5	24.1 ± 1.7**
Stem	30.1 ± 3.7	17.7 ± 1.6**
Root	41.6 ± 5.4	22.7 ± 2.8**

Notes: Biomass of WT and *lhy-10* trees shown in Fig.S2. Measures are mean ± 1SE. Student's *t*-test was used and probabilities indicated as **, $P < 0.01$, n = 12 for both genotypes.
