# Supplementary material for: Circadian clock components control daily growth activities by modulating cytokinin levels and cell division‐associated gene expression in Populus trees
Source: Plant Cell Environ. 2018 Apr 15;41(6):1468–82. doi: 10.1111/pce.13185 (PMC6001645; doi:10.1111/pce.13185)
Supplement: Supplementary file 1 — Data S1 Supporting information [file PCE-41-1468-s001.zip › Table S2_PCE.pdf]

Table S2. Estimated peak phase of rhythmic gene expression for *Populus* orthologs of core-clock genes in WT and *lhy-10* trees

Probe set	Poplar Gene Model (v3.0)	PopArray annotation	Proposed Ortholog Common	WT Phase (ZT)	*lhy-10* Phase (ZT)
PtpAffx.125536.1.S1_at	Potri.002G180800	*LHY* (AT1G01060.4)	*LHY1*	4.8	0.5
Ptp.4550.1.S1_at	Potri.014G106800	*LHY* (AT1G01060.3)	*LHY2*	3.9	0.7
PtpAffx.7997.1.S1_at	Potri.014G106800	*LHY* (AT1G01060.3)	*LHY2*	4.6	0
PtpAffx.202306.1.S1_at	Potri.002G179800	*PRR5* (AT5G24470.1)	*PRR9a*	8.0	0
PtpAffx.44182.1.A1_at	Potri.002G179800	*PRR5* (AT5G24470.1)	*PRR9a*	6.0	0
PtpAffx.18265.1.A1_at	Potri.014G106000	*PRR5* (AT5G24470.1)	*PRR9b*	8.1	0
Ptp.2304.1.A1_s_at	Potri.012G005900	*PRR5* (AT5G24470.1)	*PRR5a*	11.4	7.8
Ptp.5249.1.S1_at	Potri.012G005900	*PRR5* (AT5G24470.1)	*PRR5a*	11.0	6.7
PtpAffx.37666.1.S1_at	Potri.015G002300	*PRR5* (AT5G24470.1)	*PRR5b*	11.3	0
PtpAffx.207664.1.S1_x_at	Potri.008G046200	*PRR7* (AT5G02810.1)	*PRR7a*	12.0	10.5
PtpAffx.29932.2.S1_s_at	Potri.008G046200	*PRR7* (AT5G02810.1)	*PRR7a*	12.4	10.6
PtpAffx.29932.1.S1_at	Potri.010G215200	*PRR7* (AT5G02810.1)	*PRR7b*	13.4	10.5
PtpAffx.6319.1.S1_at	Potri.010G215200	*PRR7* (AT5G02810.1)	*PRR7b*	0	10.8
PtpAffx.6319.1.S1_s_at	Potri.010G215200	*PRR7* (AT5G02810.1)	*PRR7b*	0	10.2
PtpAffx.215744.1.S1_at	Potri.015G061900	*TOC1* (AT5G61380.1)	*TOC1*	11.9	9.7
Ptp.2473.1.A1_s_at	Potri.005G196700	*GI* (AT1G22770.1)	*GI1*	12.2	0
PtpAffx.12097.1.A1_at	Potri.008G068200	*ELF4* (AT2G40080.1)	*ELF4*	13.5	11.4

Notes: Time is indicated as ZT (h). *Populus* gene models associated with Affymetrix probe sets were identified from PopArray (version 3.0) and assigned to specific common gene names based on these annotations as well as Takata et. al., (2010) *BMC Evolutionary Biology* **10**:126
